# High-pressure macromolecular crystallography as a tool for studying protein flexibility

**DOI:** 10.1107/S2052252526008468

**Published:** 2026-10-19

**Authors:** Julia Lieske, Sofiane Saouane, Patrick Y. A. Reinke, Sven Falke, Wiebke Ewert, Jan Meyer, Tim Pakendorf, Anja Burkhardt, Eva Crosas, Johanna Hakanpää, Karolina Stachnik, Jochen Sieg, Matthias Rarey, Miriam Aßmann, Henning Zaun, Jürgen Kuballa, Georgii K. Selikhanov, Azat G. Gabdulkhakov, Henry N. Chapman, Sebastian Günther, Alke Meents

**Affiliations:** a Center for Free-Electron Laser Science CFEL Deutsches Elektronen-Synchrotron DESY Notkestraße 85 Hamburg22607 Germany; b Deutsches Elektronen-Synchrotron DESY Notkestraße 85 Hamburg22607 Germany; c ZBH - Center for Bioinformatics Universität Hamburg Bundesstraße 43 Hamburg20146 Germany; d GALAB Laboratories GmbH Am Schleusengraben 7 Hamburg21029 Germany; e Institute of Protein Research Russian Academy of Sciences Pushchino Moscow Region142290 Russian Federation; f Department of Physics Universität Hamburg Luruper Chausee 149 Hamburg22761 Germany; g Centre for Ultrafast Imaging CUI Universität Hamburg Luruper Chaussee 149 Hamburg22761 Germany; University of Virginia, USA

**Keywords:** high-pressure crystallography, sapphire cells, proteins under pressure, protein dynamics, protein structure, X-ray crystallography

## Abstract

Protein function depends on their conformational flexibility. Complementary to temperature-dependent studies, high-pressure (HP) macromolecular crystallography is well suited to exploring the conformational space of proteins at atomic resolution. We have developed a new HP cell concept for macromolecular X-ray crystallography that overcomes the limitations of current devices. It consists of a single-crystalline sapphire capillary, which is loaded with multiple crystals and pressurized with helium at freely adjustable gas pressures of up to 120 MPa. Studying five different proteins, we generally observed a compression of the protein structures and a decrease in thermal displacement parameters at high pressure. Structurally, observations included the compression of a central tunnel of a biotechnologically relevant enzyme and the stabilization of ligand binding in the membrane protein photosynthetic reaction center of *Cereibacter sphaeroides*. For two pharmaceutically relevant proteins, the human kinase DRAK2 and SARS-CoV-2 main protease, we identified concerted movements of subdomains. Such knowledge is essential for predictions of protein dynamics and defined conformational states, offering the potential for improved structure-based drug development.

## Abstract 2

A new concept for high-pressure macromolecular crystallography is presented and applied to five diverse proteins.

## 1. Introduction

Proteins fulfill various functions in the cell, such as performing enzymatic reactions, transducing signals via allosteric pathways, transporting molecules, or regulating genes by binding to DNA. The functionality of a protein relies on a well defined degree of flexibility, the so-called conformational space, which is inherently encoded in the three-dimensional structure of the protein. This conformational space is not only determined by the biochemical environment of the protein, *i.e.* the presence or absence of certain ligands or substrates, its solvents or other protein binding partners, but is also altered by physical parameters such as temperature and pressure (Winter, 2019 ▸). X-ray crystallography is a powerful technique to determine protein structures and investigate protein dynamics (Rhodes, 2006 ▸; Afonine *et al.*, 2025 ▸). Multi-temperature diffraction experiments conducted between 100 K and room temperature provide mechanistic insights into protein function by revealing various intermediate states indicated to be functionally relevant (Keedy *et al.*, 2015 ▸; Fischer *et al.*, 2015 ▸; Günther *et al.*, 2021 ▸; Stachowski & Fischer, 2026 ▸). Experiments at elevated pressures also allow insights into intermediate states (Fourme *et al.*, 2012 ▸). Depending on the applied pressure, effects on proteins range from cavity volume changes, altered substrate binding, changes in catalytic turnover rates, shifts in the enantioselectivity of enzymes, dissociation of oligomeric complexes, or even denaturation by unfolding of the protein in extreme cases (Winter & Dzwolak, 2004 ▸; Eisenmenger & Reyes-De-Corcuera, 2009 ▸; Pattni & Heyden, 2019 ▸; Prangé *et al.*, 2022 ▸; Akasaka, 2025 ▸). Hence, HP studies of protein structures have the potential to elucidate details of enzymatic reaction mechanisms and protein unfolding processes, or can answer the question of how proteins adapt to natural HP habitats such as the deep sea or the Earth’s crust (Sieg *et al.*, 2022 ▸). This knowledge ultimately enables several HP applications, such as the design of pressure-adapted enzymes for optimized performance in biotechnological processes and food processing at ≥100 MPa (Prangé *et al.*, 2022 ▸; Yamamoto, 2017 ▸).

Unlike temperature-dependent studies, experiments at elevated pressures are more demanding due to technical challenges, such as the pressure stability of the equipment and the achievable signal-to-noise ratio limited by signal transmission through the sample containment – although the state of the art is constantly improving. In addition to X-ray crystallography, current methods used in HP studies involve various spectroscopic and scattering techniques including NMR spectroscopy (Winter & Dzwolak, 2004 ▸; Collins *et al.*, 2011 ▸). Whereas devices for HP NMR spectroscopy are currently limited to a few hundred megapascals, diamond anvil cells (DACs) commonly used in HP X-ray crystallography can generate pressures of hundreds of gigapascals as they are designed for experiments under extreme conditions in geo­logical and materials sciences (Kurpiewska *et al.*, 2023 ▸). HP cryocooling of protein crystals at pressures of up to 500 MPa is another approach in HP crystallography, primarily developed to avoid the need for cryoprotectants (Kim *et al.*, 2005 ▸; Burkhardt *et al.*, 2012 ▸). Despite the variety of different experimental techniques, as of January 2026 only approximately 30 individual proteins had been studied at pressures higher than 20 MPa and approximately 230 structures had been deposited in the Protein Data Bank (Berman *et al.*, 2003 ▸) (https://wwpdb.org; see Table S5 in the supporting information for details of pressure-related depositions). A wider application is probably hindered by technical limitations of current methods.

Commonly used DACs possess several disadvantages for the measurement of biological samples such as protein crystals. Crystal loading into a DAC is time consuming, sensitive crystals are potentially damaged during loading and pressurization, and they typically provide space for very few crystals, thereby hampering structure determinations from low-symmetry crystals (Kurpiewska *et al.*, 2023 ▸). Further, it is difficult to adjust and measure accurately the pressure inside the DAC at moderate pressures relevant for biological samples, as the typically applied monitoring of the fluorescence shift of a ruby chip inside the DAC is temperature dependent and the derived pressure values are only reliable at pressures above 1 GPa (Ragan *et al.*, 1992 ▸; Grasset, 2001 ▸). To overcome these practical challenges of DACs, we developed a new cell concept for HP macromolecular crystallography experiments in the biologically relevant pressure range of 0.1 to 120 MPa, the latter corresponding to the pressure in the deep sea 12 kilometres below sea level. The device is based on a thin-walled sapphire capillary in combination with a fixed-target sample holder, using helium gas as the pressurizing agent.

Here we present the results of crystallographic studies of five different proteins measured at pressures up to 120 MPa using our new HP cell. We demonstrate that HP crystallography with this cell concept enables the investigation of scientifically relevant macromolecular crystals, including membrane proteins, and small and fragile crystals of low symmetry such as the SARS-CoV-2 main protease (M^pro^). Our experiments allowed us to identify concerted movements of subdomains, reflecting inherent protein flexibility, as well as altered ligand binding.

## 2. Materials and methods

### 2.1. HP setup and HP cell for macromolecular crystallography experiments

The new multi-crystal HP crystallography concept essentially consists of two main components: the pressure cell itself, which contains the samples, and a two-stage pressure booster that supplies the appropriate gas pressure for the experiments. The two components are connected by very thin and therefore flexible pressure-resistant capillaries.

**Figure 1:**
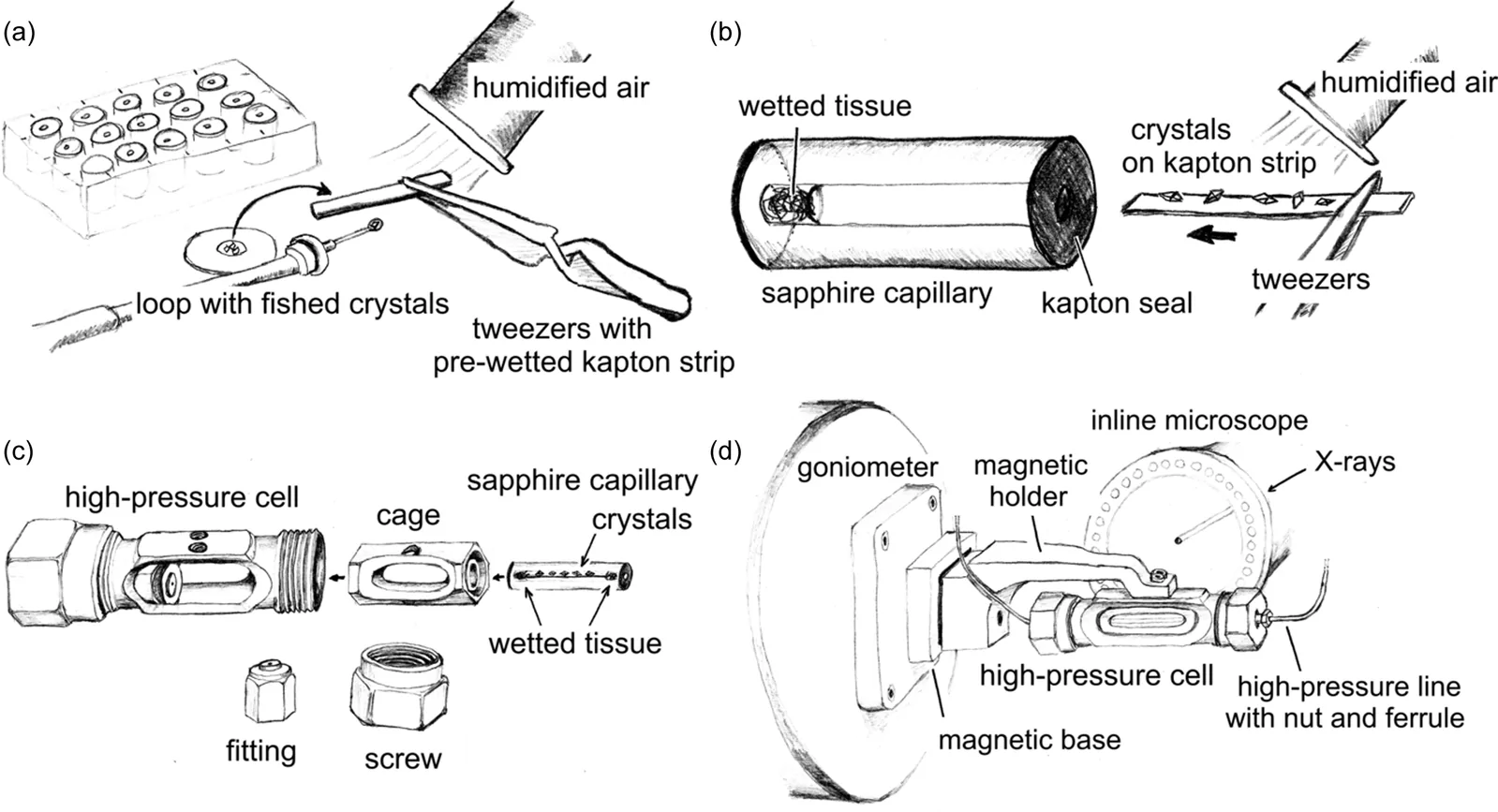
General procedure of an HP experiment with the cell. (*a*) Multiple protein crystals are transferred onto a pre-wetted Kapton support strip (1100 × 650 × 75 µm) in a stream of humidified air to prevent crystal dehydration. The application of protective oil (*e.g.* NVH oil) is recommended at this stage for dehydration-sensitive crystals. (*b*) The Kapton strip is inserted into the sapphire capillary, which already contains a small piece of wet paper tissue serving as a liquid reservoir at the opposite end. Following the Kapton strip, a second piece of paper tissue is inserted into the capillary from the same side, which is then immediately soaked with reservoir solution. (*c*) Subsequently, the capillary with the crystals is inserted into the central steel cage of the HP cell. The capillary with the Kapton strip can now be manually rotated to orient the crystals favorably with respect to the opening window of the cell and point towards the inline sample-viewing microscope when mounted later on the goniometer. Now, the cell assembly is completed and the screws are tightened. (*d*) The HP cell is attached to the goniometer of the beamline using a magnetic holder and the crystals are optically centered in the 26 keV X-ray beam. Prior to data collection, the cell is pressurized via nickel-clad fused-silica lines on both sides, which are connected to a double-stage air-driven helium compressor.

The pressure cell was designed and manufactured by suna-precision GmbH. The basic design (Fig. 1 ▸) consists of a sapphire capillary that is clamped into a holder and can be pressurized from both sides via connected high-pressure capillaries. The decision to use a capillary as a pressure cell arose because other geometries – such as a blind hole – were technically unfeasible or would have resulted in excessive internal stresses. Single-crystal sapphire was selected as the material for the capillary because, on the one hand, it is colorless and optically transparent, allowing sample centering to be performed with the on-axis microscope on the beamline, and, on the other hand, because sapphire capillaries can be manufactured as a single-crystalline material, so that only a few Bragg reflections from the capillary material itself are generated which would interfere with the diffraction pattern of the sample. Furthermore, aluminium and oxygen have comparatively low absorption cross sections for X-rays, meaning that the comparatively weak Bragg reflections from the protein crystals are only slightly attenuated. Pictures of the HP cell parts and technical details are provided in the supplementary information (Figs. S5 and S6). The capillaries used typically have an inner diameter of about 0.6 mm and an outer diameter of 3 mm. In our experiments, the capillaries could routinely withstand pressures of more than 120 MPa. For loading of the crystals, they are first placed on a Kapton sample holder, which is then inserted into the capillary from the side. To prevent the crystals from drying out during this process, these steps are performed in an atmosphere with controlled high humidity. Next, the cell, which has already been loaded with the samples, is assembled, and the capillaries for subsequent pressurization are connected on both sides. The detailed procedure of crystal loading into the HP cell and cell assembly is depicted in Fig. 1 ▸. After loading, the cell is installed on the goniometer of the beamline using a specially designed magnetic holder.

The cell can then be pressurized with helium gas, which is applied through the two high-pressure capillaries connected to both ends of the sapphire capillaries. Helium gas was chosen as the pressurizing medium as it is supposed not to interact with protein crystals, it has a low absorption and scattering cross section for X-rays, and it does not undergo any phase transition in the chosen temperature and pressure range. To keep helium consumption for the experiments as low as possible and to ensure a high level of safety, the entire pressurized system was designed with a very small volume. In the event of an accident, therefore, only extremely small amounts of helium would be released with little kinetic energy, minimizing the impact on equipment and people.

For generating helium gas with pressures of up to 240 MPa we use a double-stage air-driven compressor station from Maximator GmbH. The compressor station automatically re-adjusts the preset helium pressure level in case of pressure loss due to potential leakages in the experimental setup. Two commercially available pressure sensors with calibrated digital output are installed at the compressor itself and next to the HP cell (BD sensors DMP and WIKA HP-2), allowing for continuous *in situ* pressure monitoring and logging of the setup status during the experiment. The applied pressure is freely adjustable between atmospheric pressure and 120 MPa with a precision of a few megapascals. A remotely controlled three-way air-actuated HP valve (VICI/VALCO) between the compressor and the HP cell allows controlled pressurization and decompression of the cell. All parts of the setup are connected via commercially available 1/32-inch UHPLC nickel-clad fused silica tubing with small inner diameters between 50 and 180 µm and by standard HP 1/4-inch steel tubing. Connections between different tubing types are accomplished via custom-made adapters (Maximator GmbH/VICI). A schematic drawing of the complete experimental setup is shown in Fig. S7.

### 2.2. Data collection and processing

All X-ray diffraction data were collected on the P11 beamline of the PETRA III storage ring at DESY in Hamburg (Burkhardt *et al.*, 2016 ▸). Further details of the experimental setup are shown in Figs. S8 and S9. The standard graphical user interface of P11 was used, as in a conventional macromolecular crystallography experiment. Customized software limits for the goniometer were applied to avoid collisions of the HP cell with parts of the beamline, *e.g.* the collimator. The software environment allows for optical and X-ray-assisted crystal centering and an automated data processing pipeline via *XDSAPP* (Sparta *et al.*, 2016 ▸), providing fast feedback on crystal quality, unit-cell constants and data completeness. Diffraction data were recorded at a photon energy of 26 keV to minimize sapphire absorption and with an X-ray beam size of 50 × 50 µm (FWHM, H×V) at the sample position on a Pilatus3 X 2M CdTe detector. Data were collected in 45° rotation wedges at each crystal position. To collect data at different pressures from the same crystal, crystals were exposed multiple times at different pressures, each at a new position. Radiation damage was avoided by dose estimation via the ‘expected crystal lifetime calculator’ (https://bl831.als.lbl.gov/xtallife.html) and by applying an offset of at least 50 µm for multiple illuminations of the same crystal.

All data were manually reprocessed using *XDS* (Kabsch, 2010 ▸) and suitable data wedges were merged via *XSCALE* (Kabsch, 2010 ▸) to obtain complete data sets for each data point. No absorption correction was applied for the intensities (Ahsbahs, 2004 ▸). Possible shadowing of the diffraction images caused by metal parts of the HP cell was checked by manual inspection of the background images delivered by *XDS* and was accounted for during processing by background adaptation.

### 2.3. Protein crystallization

Trigonal crystals of ribonuclease A from *Bos taurus* (Sigma, R5500) were grown by the hanging drop vapor diffusion method as described previously by Zegers *et al.* (1994 ▸). Briefly, 10–20 mg ml^−1^ protein dissolved in 0.1 *M* sodium acetate pH 4.5 were mixed in a 1:1 ratio with precipitant containing 0.1 *M* sodium acetate pH 4.5, 1.2–1.5 *M* ammonium sulfate and 2–2.5 *M* sodium chloride, and equilibrated for one to two days at room temperature to obtain 200 µm sized crystals.

The human serine/threonine kinase 17b, also known as DRAK2, was purified and crystallized in batch in the presence of the inhibitor quercetin as described earler (Lieske *et al.*, 2019 ▸). To obtain the DRAK2-ATP/ADP complex, the 50–75 µm sized batch-grown crystals were subsequently mixed in a 1:1 ratio with 100 m*M* ATP in DRAK2 crystallization buffer (0.1 *M* HEPES pH 6.5, 0.2 *M* ammonium acetate, 20% PEG 3350, 50 m*M* sodium/potassium tartrate, 50 m*M* magnesium chloride) and incubated for half an hour before they were used for HP diffraction experiments.

SARS-CoV-2 M^pro^ was purified and crystallized as described previously (Günther *et al.*, 2021 ▸). Due to their 200 × 150 × 20 µm plate-like shape, M^pro^ crystals are prone to dehydration and hence were protected by application of NVH oil at the HP cell loading step.

*Pedobacter heparinusN*-acetyl­glucosamine 2-epimerase (AGE) was overexpressed in *Escherichia coli* and purified as described (Reich *et al.*, 2022 ▸). Crystallization conditions for AGE were optimized based on the first screening hit, LMB Crystallization Screen (Molecular Dimensions) well G4. The best crystallization results were obtained at 12.6 mg ml^−1^ protein in 20 m*M* sodium phosphate buffer pH 7.5 mixed (4+2 µl) with 0.1 *M* sodium cacodylate buffer pH 5.0, 0.35 *M* sodium acetate and 26% PEG8000, leading to 70 × 400 × 700 µm plate-shaped crystals at room temperature after 5–7 days.

For purification of the F(M197)H mutant of the photosynthetic reaction center of the purple bacterium *Cereibacter sphaeroides* we used the protocol described previously (Fufina *et al.*, 2007 ▸). Crystallization conditions were applied as described (Wöhri *et al.*, 2009 ▸) with minor modifications. Batch crystallization was chosen instead of vapor diffusion to increase the number of grown crystals. RC solution (20–25 mg ml^−1^) was mixed with LSP in a 1:1 ratio. Around 20–30 µl of this mixture were placed into a sealed plastic tip and covered with an excess amount of 1 *M* tri­sodium citrate which acted as precipitant. Crystal growth took about one week at 21°C in the dark.

### 2.4. Structural analysis

For structure solution via molecular replacement with *Phaser* (McCoy *et al.*, 2007 ▸), PDB entries 2e3w (Boerema *et al.*, 2008 ▸), 6qf4 (Lieske *et al.*, 2019 ▸), 2gz6 (Lee *et al.*, 2007 ▸), 7ar5 (Günther *et al.*, 2021 ▸) and 3v3y (Vasilieva *et al.*, 2012 ▸) were used as templates for RNase A, DRAK2, AGE, M^pro^ and the RC, respectively. Structure solution was followed by iterative cycles of refinement in *Phenix* (Afonine *et al.*, 2012 ▸; Liebschner *et al.*, 2019 ▸) and manual model building in *Coot* (Emsley *et al.*, 2010 ▸).

Crystal packing analysis, *B*-factor analysis and r.m.s.d. calculations were done with the CCP4 modules *PISA*, *baverage* and *superpose* (Potterton *et al.*, 2003 ▸; Krissinel & Henrick, 2007 ▸, 2004 ▸). Pressure-induced intramolecular movements were estimated via C_α_—C_α_ distance calculations and images were prepared using *PyMOL* (*The PyMOL Molecular Graphics System*, Version 1.8.4.0, Schrödinger LLC). Protein void volume analysis was done with the *ProteinVolume 1.3* software using a volume probe radius of 0.02 Å and a surface probe minimum distance of 0.1 Å (Chen & Makhatadze, 2015 ▸). Difference distance matrices were calculated in *Chimera* (Chen *et al.*, 2015 ▸) and their presentation optimized in *Origin* (OriginLab). Hinge movement analysis of DRAK2 domains was performed via the domain selection routine of the *DynDom* web server, which is designed to analyze concerted domain movements (Girdlestone & Hayward, 2016 ▸). *DynDom* domain rotations were calculated in *PyMOL* via the *angle_between_domains* plugin. For better comparison, hydrogen-bond networks for each structure were computed using *Protoss* (Bietz *et al.*, 2014 ▸) and the *Naomi* library (Urbaczek *et al.*, 2011 ▸) to determine the protonation states and hydrogen-bond interactions.

## 3. Results

### 3.1. HP structure determinations

We performed HP structure determinations of five different proteins: (i) ribonuclease A (RNase A) from *Bos taurus*, a well studied model protein previously used for HP studies up to 0.67 GPa (Kurpiewska *et al.*, 2016 ▸), (ii) the *Pedobacter heparinus* AGE, one of two enzymes used for the two-stage industrial synthesis of sialic acid where pressure potentially increases reaction efficiency (Reich *et al.*, 2022 ▸), (iii) the *Cereibacter sphaeroides* photosynthetic reaction center (RC) as a representative of membrane proteins, (iv) the human serine/threonine kinase 17B (DRAK2), a cancer-related pharmaceutically relevant target protein (Sanjo *et al.*, 1998 ▸; Doherty *et al.*, 2009 ▸; Edwards *et al.*, 2015 ▸; Szoltysek *et al.*, 2020 ▸), and (v) the SARS-CoV-2 M^pro^, an essential cysteine protease of SARS-CoV-2 (Günther *et al.*, 2021 ▸). Crystal structures were determined at pressures of up to 120 MPa, yielding maximum resolutions of 1.5 Å, 1.7 Å, 2.5 Å, 2.6 Å and 2.35 Å for RNase A, AGE, the F(M197)H mutant of the RC, DRAK2 and M^pro^, respectively. Data collection and refinement statistics are provided in Tables S1–S4.

**Figure 2:**
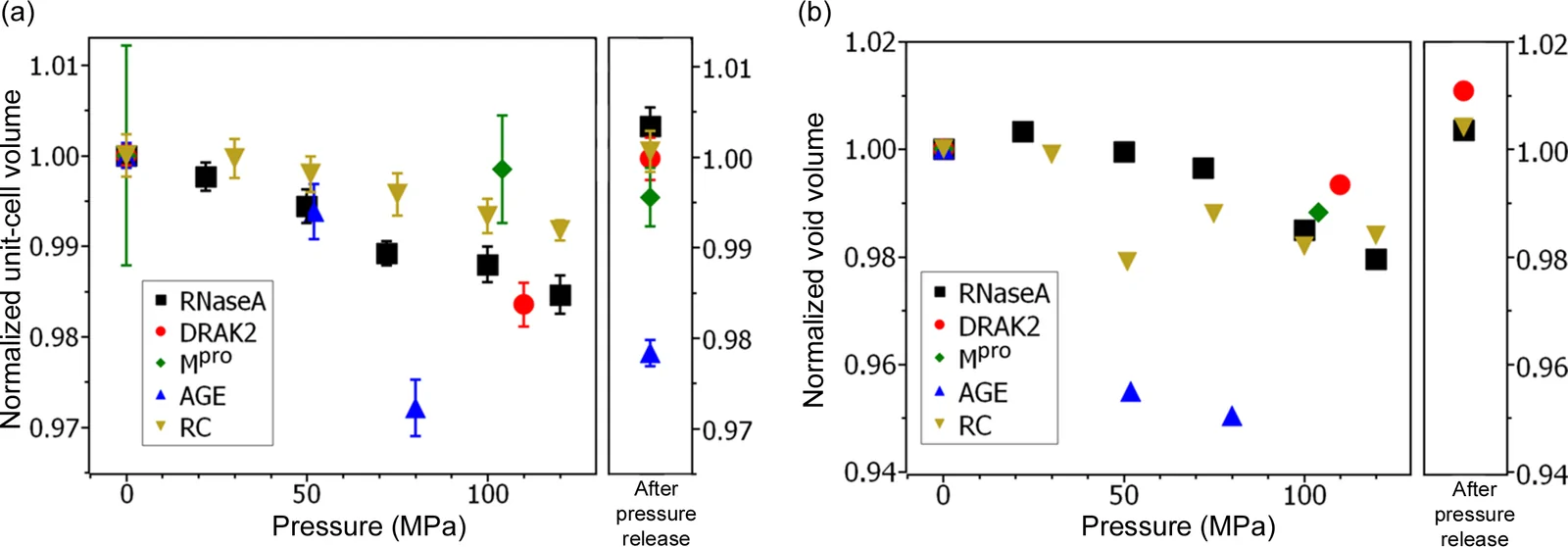
Changes in (*a*) unit-cell volumes and (*b*) void volumes of the five studied proteins at different pressures. All values are normalized to the corresponding values at atmospheric pressure. The depicted unit-cell volumes are the normalized average volumes calculated from 2–19 individual crystals, with the error bars displaying the normalized mean of the estimated standard deviation of the unit-cell volume. Void volumes were calculated from the refined structures using *ProteinVolume 1.3* (Chen & Makhatadze, 2015 ▸). Due to the incompleteness of the data sets, there are no void volume values for M^pro^ and AGE after pressure release.

The unit-cell parameters of the crystals of all five proteins decrease with increasing pressure. The compression of the unit-cell volumes varies significantly between the investigated proteins. While M^pro^ crystals showed the smallest compression of only 0.15% at a pressure of 104 MPa compared with ambient conditions, the largest compression of the unit-cell volume by 2.8% was observed for AGE crystals already at 80 MPa. The unit-cell volumes of other protein crystals were compressed by 0.7% (RC, 100 MPa), 1.2% (RNaseA, 100 MPa) and 1.6% (DRAK2, 110 MPa) [Fig. 2 ▸(*a*)]. Upon pressure release, the unit-cell parameters of RNase A, DRAK2, M^pro^ and the RC returned to their initial values, whereas the AGE crystals did not fully revert. Generally, we see a trend towards stronger unit-cell compression with lower solvent content of the crystal, although this is not observed in the case of M^pro^ (Fig. S10).

### 3.2. HP effects on protein structures

For all five studied proteins we did not observe significant changes in the overall three-dimensional folds upon pressurization. Generally, structural analysis shows that the atom displacements caused by approximately 100–120 MPa pressure are subtle and tend not to exceed 1 Å. However, as these changes involve not only individual residues but commonly whole regions ranging from secondary structure elements up to entire domains, we consider them to be significant.

**Figure 3:**
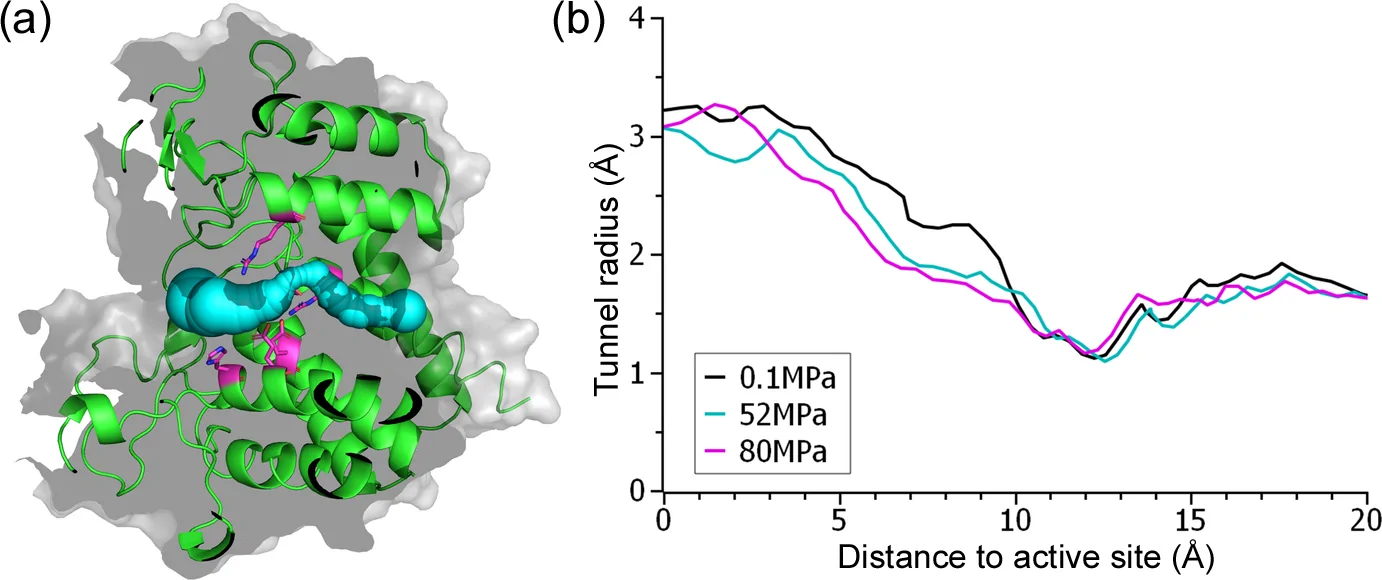
Pressure effect on AGE from *Pedobacter heparinus*. AGE consists of a homodimer of two (α/α)_6_-barrels in a ‘front-to-front’ configuration. (*a*) The cut through of one AGE monomer is shown in both the surface representation (gray) and the cartoon representation (green), allowing visualization of the shape of the central tunnel highlighted as blue spheres. Putative active site residues in close proximity (His244, His378, Glu314, Arg63, Glu247 and Arg381) are presented as pink sticks. (*b*) Parts of the central tunnel of the (α/α)_6_-barrel structure of AGE are compressed at 52 MPa (cyan line) and 80 MPa (pink line) compared with the atmospheric pressure structure (black line).

High pressure is able to deform and ultimately unfold protein structures. Mechanistically this process involves the collapse of small cavities, so-called solvent-excluded void volumes, inside the protein (Collins *et al.*, 2011 ▸). Consistent with this, we observed a reduction in the void volumes for all five protein structures [Fig. 2 ▸(*b*)], with the void volume defined as the difference between the solvent-excluded volume and the invariable van der Waals volume of the protein (Chen & Makhatadze, 2015 ▸). Void volumes decreased by 0.7% for DRAK2, 1.2% for M^pro^, 1.5% for RNase A, 1.8% for the RC and 5.0% for AGE at 80–110 MPa. Of all five studied proteins, AGE exhibits the least densely packed structure and hence has the largest potential for void volume compression – packing density being defined as the ratio of van der Waals volume to solvent-excluded volume. Additionally, we observed a reduction in the radii of the central tunnels of AGE, which consists of a dimer of two (α/α)_6_-barrels (Fig. 3 ▸).

### 3.3. Concerted pressure-induced movements correlate with protein architecture

**Figure 4:**
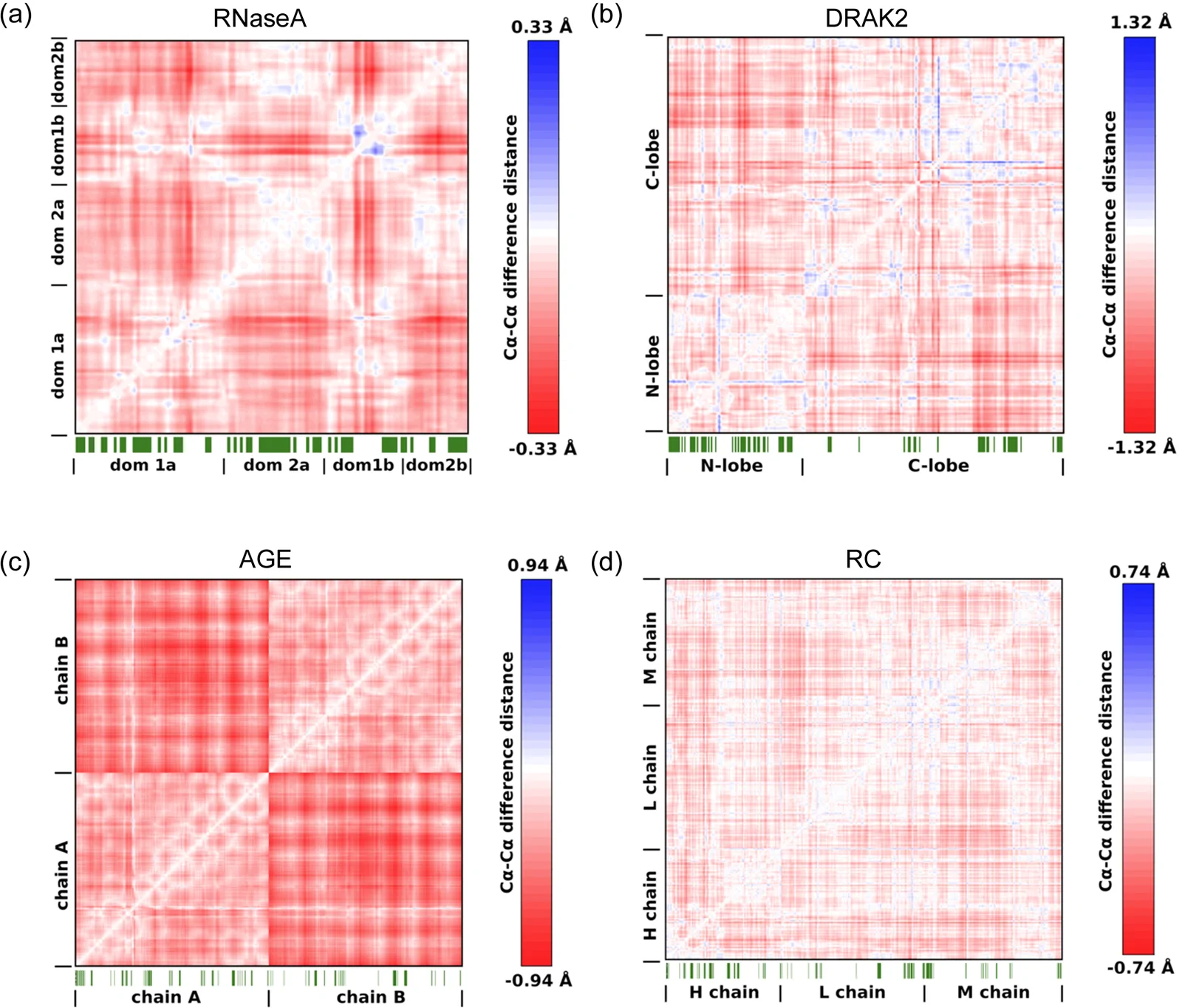
C_α_—C_α_ DDMs showing the pressure-induced compression of the structures. Shown are (*a*) RNaseA at 120 MPa, (*b*) DRAK2 at 110 MPa, (*c*) AGE at 80 MPa and (*d*) the RC at 120 MPa compared with the corresponding structures at atmospheric pressure. Domains are labeled for a better overview. Negative values indicate compression. Maximum compression values differ between protein systems. Maximum values for each system were used for individual color scale definition for clarity. Crystal contacts of the HP structures are indicated as green line patterns below the matrices. Indicated are all interfacing crystallography-related residues according to *PISA* (Krissinel & Henrick, 2007 ▸).

The observed pressure-induced atomic movements are structure-related, as they do not show a linear compression of the structure but contain information on pressure-induced shifts of domains or secondary structure elements and hence depend on the individual architecture of the studied protein. In this regard, RNase A and DRAK2 exhibit strong similarities because both enzymes are comprised of two domains divided by a large cleft containing the active site. Whereas the RNase A structure is very pressure-resistant and only showed minor changes, the difference distance matrices (DDMs) of DRAK2 comparing the HP and ambient-pressure structures reveal significant collective movements of adjacent residues [Fig. 4 ▸(*b*)]. The C_α_—C_α_ difference distances within a domain (0.08–0.10 Å) are smaller on average than between different domains (0.21 Å). This phenomenon gives rise to the appearance of block patterns in the DDM that are characteristic for the domain structure of the respective protein. Overall, the observed changes are relatively small and in many cases fall within the coordinate uncertainty as reported by the refinement program (Lunin *et al.*, 2002 ▸). Nevertheless, regions with movements above the combined uncertainty for the compared structures were identified for all proteins except RNaseA (Fig. S11).

*Pedobacter heparinus* AGE consists of a dimer of two (α/α)_6_-barrels in a ‘front-to front’ configuration, as the *PISA* analysis clearly reveals. It does not contain domains that can swing in a hinge-like movement as is the case for DRAK2. The monomers were rather uniformly compressed and moved towards each other by approximately 0.5 Å at 80 MPa pressure, as estimated by the distance between their centers of mass. The dimer interface area was increased from 9.0% to 9.4%, and additionally most hydrogen bonds and salt-bridge interactions at the interface were shortened by 0.1–0.2 Å, ultimately leading to a strengthening of the dimer interface. Comparable with the results for DRAK2, the C_α_—C_α_ DDM of AGE shows block patterns [Fig. 4 ▸(*c*)]. These patterns indicate that, at the applied pressure, the displacements of the monomers *A* and *B* towards each other were larger than the compression within each monomer. The structure of individual α-helices is clearly visible as a regular checked pattern in the DDM.

### 3.4. Pressure effects on a membrane protein

To the best of our knowledge, the *Cereibacter sphaeroides* photosynthetic reaction center (RC) is the first membrane protein structure studied at high pressure, although the stability of the hydrogen bonds of the special pair in the RC has been investigated under pressure using absorption spectroscopy (Kangur *et al.*, 2017 ▸). The RC is a relatively large (∼100 kDa) transmembrane pigment–protein complex consisting of three polypeptide chains (L, H and M). It has no enzymatic activity but forms a scaffold for its ten non-covalently bound cofactors, *i.e.* four bacterio­chlorophylls, two bacteriopheophytins, two quinones, one non-heme Fe^2+^ ion and one carotenoid molecule (Leonova *et al.*, 2011 ▸). We observed that high pressure generally induces a reversible, subtle and uniform compression of the protein complex [Figs. S3(*a*) and S3(*b*)]. Nevertheless, we also observed concerted shifts of adjacent residues in the DDM of the RC, albeit on a smaller scale than for DRAK2 and much more complex due to the larger size of the protein [Fig. 4 ▸(*d*)]. The H chain is clearly divided into two subdomains (H10–130 and H131–249). The first subdomain consists of a long N-terminal helix and an ensemble of surface loops, whereas the second subdomain is a rather compact arrangement of β-strands and two helices situated on top of the central bundle of transmembrane helices.

In the active RC the electron transfer from the primary to the secondary quinone is associated with proton transport, and hence the conversion of light energy into chemical energy (Breton *et al.*, 1994 ▸). The quinone at the Q_B_ site is weakly bound and leaves the RC after accepting two protons and two electrons, in this way functioning as an essential proton transporter in photosynthesis. In our structures, the ligand at this site is not the naturally occurring quinone but was exchanged with a monoolein due to the crystal preparation procedure (Selikhanov *et al.*, 2020 ▸, 2022 ▸). However, both molecules share their long aliphatic tail and the monoolein is positioned in such a way as to interact with l-Ser223 and l-Asp213 as well, both being crucial residues responsible for the final proton transfer to the acceptor quinone.

At 120 MPa, the *B* factors of the RC were decreased on average by 8.4 Å^2^ for all protein atoms and by 12.5 Å^2^ for the atoms of the ten non-covalently bound cofactors, with the largest decrease of 25.5 Å^2^ observed for the monoolein molecule bound to the Q_B_ site [Fig. S3(*c*)]. As proven by the additional 2*mF*_o_–*DF*_c_ electron density present for the monoolein molecule in the structures determined above 100 MPa, the long tail of this ligand is less delocalized at high pressure and two distinct conformations of the tail become more populated (Fig. S4). In addition to the *B*-factor decrease, this indicates a pressure-induced reduction in flexibility and hence stabilization of the ligand binding to the Q_B_ site.

### 3.5. Kinases – comparing pressure-induced movements with biological function

**Figure 5:**
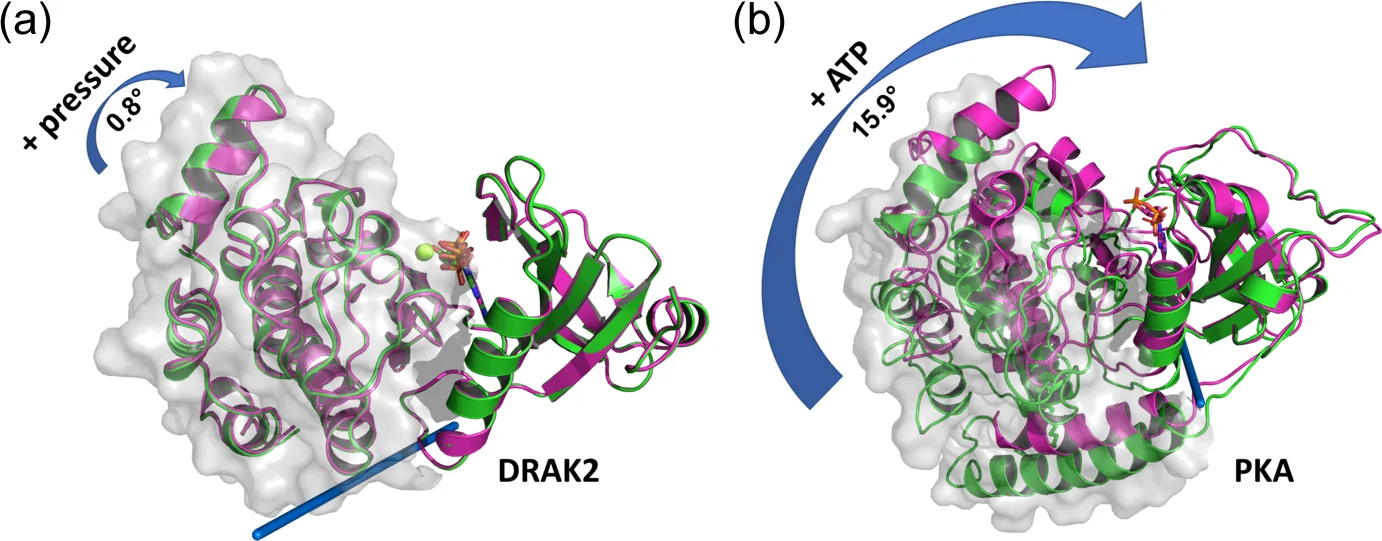
Domain movements in kinases. Comparison of (*a*) the pressure-induced domain movement of DRAK2 (by 0.8°) and (*b*) the domain movement observed upon ATP binding to the mammalian cAMP-dependent protein kinase A (PKA) (by 15.9°). Central cleft closure is observed upon ATP binding to PKA and – to a much smaller extent – upon pressure perturbation in DRAK2. The structures at atmospheric pressure and the corresponding nucleotide-free form are shown in green, whereas the 110 MPa structure and the ATP-bound state are colored pink. The structures are superimposed on their N-lobes (right-hand domains) and the C-lobes of the reference states are highlighted as light-gray surfaces. The rotation axes of the hinge movements as determined via the *DynDom* server (Girdlestone & Hayward, 2016 ▸) are represented as blue rods. PKA structures 1cmk (free form) and 1atp (ATP-bound form) (Zheng *et al.*, 1993 ▸) were taken from the Protein Data Bank.

The largest pressure-induced structural changes of all five studied proteins were observed for the pharmaceutically relevant target proteins DRAK2 and M^pro^. Analysis of the DRAK2 structures reveals that the domains separated by a wide cleft are closer to each other by 0.4 Å at 110 MPa, measured as the difference distance of the centers of mass of the individual domains. This is accompanied by a 0.8° rotation of the C-lobe towards the N-lobe, with the rotation axis situated close to the DRAK2 hinge region [Fig. 5 ▸(*a*)]. Regarding the direction, the pressure-induced movements observed for DRAK2 resemble the naturally occurring active site cleft closure found also in other kinases such as the cAMP-dependent protein kinase (PKA) [Fig. 5 ▸(*b*)], which plays a role in cell signaling processes and is used as a model system for this kind of serine/threonine protein kinases (Johnson *et al.*, 2001 ▸). However, the compared DRAK2 structures belong to the same nucleotide state and the observed pressure-induced changes are on a much smaller scale than the movements observed for PKA upon ATP binding. The results show which movements are generally possible for DRAK2 in the crystalline context [Fig. 4 ▸(*b*)].

### 3.6. Pressure-induced movements of SARS-CoV-2 M^pro^

The protease M^pro^, one of two viral cysteine proteases essential for the maturation of proteins involved in viral replication, is a major target for drug development against SARS-CoV-2 (Günther *et al.*, 2021 ▸; Douangamath *et al.*, 2020 ▸). Based on the high structural conservation of M^pro^ homologues among different pathogenic Betacoronaviruses (Zhang *et al.*, 2020 ▸), our findings on the SARS-CoV-2 enzyme are assumed to be valuable for analyzing the structural dynamics of those closely related proteases as well. M^pro^ consists of a homodimer, with each monomer subdivided into the N-terminal domain I, the central domain II and, linked via the long flexible P5 loop, the α-helical C-terminal domain III. The active site pocket with the catalytic cysteine 145 is situated in a cleft between the N-terminal domain I and the central domain II.

**Figure 6:**
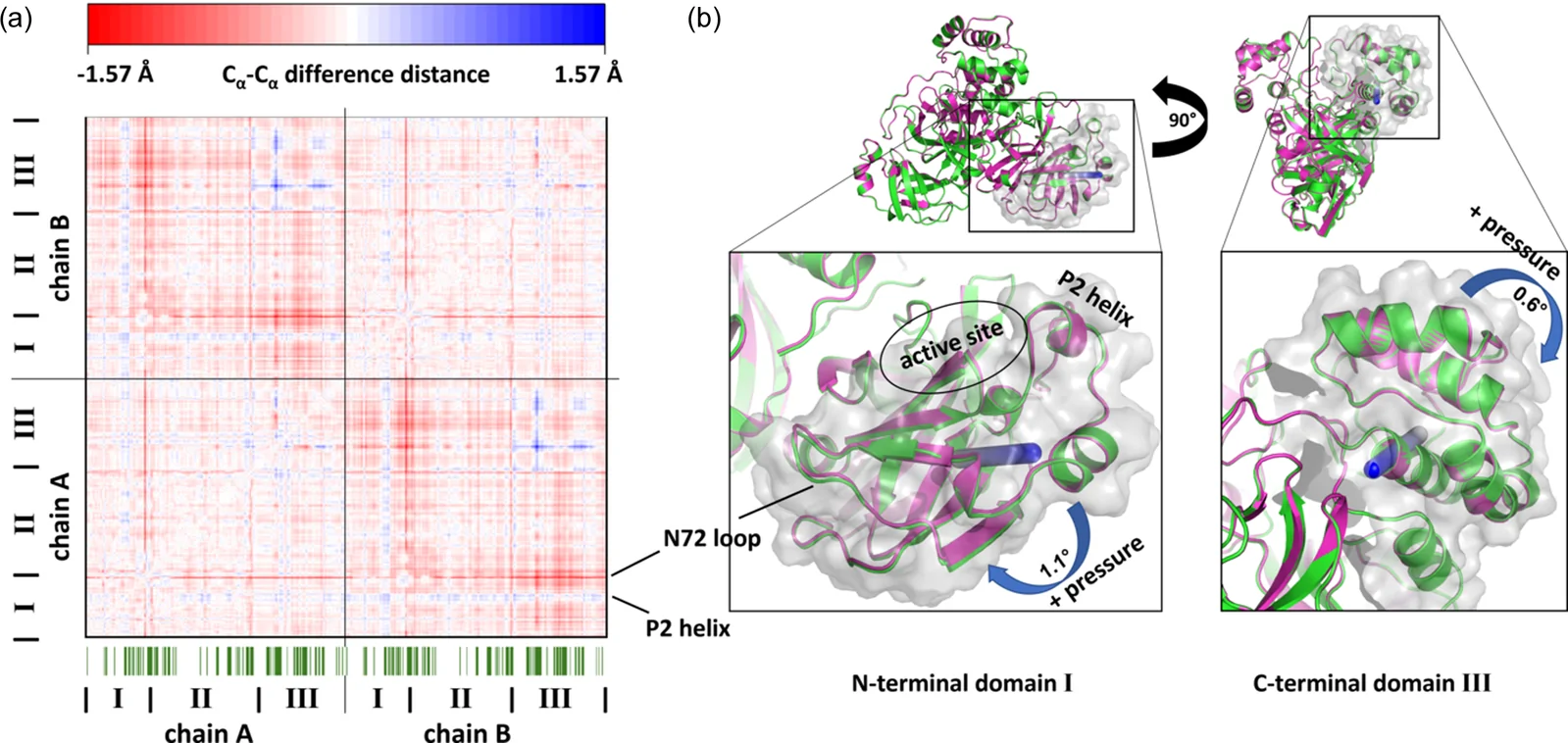
Pressure-induced movements in SARS-CoV-2 M^pro^. (*a*) C_α_—C_α_ DDM between the SARS-CoV-2 M^pro^ structures at atmospheric pressure and at 104 MPa. Crystal contacts of the HP structure are indicated as a green line pattern. One chain of the dimer contains 305 residues. Indicated are all crystallography-related interfacing residues according to *PISA* (Krissinel & Henrick, 2007 ▸). (*b*) Depiction of the observed rotation of the N-terminal domain (1.1°) and the C-terminal domain (0.6°) of M^pro^ at 104 MPa (pink) compared with atmospheric pressure (green). The structures are superimposed on the rigid parts of the central β-sheet at the dimer interface.

We solved the M^pro^ HP crystal structure at 104 MPa to 2.35 Å resolution and analyzed the pressure-induced movements compared with the structure at atmospheric pressure solved at 2.05 Å resolution. Upon structure superposition via rigid parts of the central β-sheet, we observed that high pressure induces slight rotations of both the N-terminal domain I by 1.1° and the C-terminal domain III by 0.6°. The movements in domain I involve, most importantly, the P2 helix with the adjacent loop next to the active site (45–54) by up to 0.52 Å (Leu50 C_α_) and region 62–81 with a maximum C_α_ movement of 1.1 Å observed for the loop residue Asn72. In the C_α_—C_α_ DDM these changes are clearly visible as one blue and one red stretch, respectively [Fig. 6 ▸(*a*)]. For both regions we hence confirm earlier findings describing them as flexible parts of M^pro^ (Pearce *et al.*, 2017 ▸; Ebrahim *et al.*, 2022 ▸). Notably, the P2 helix forms part of the active site pocket, and Met49 in particular was shown to play a major role in substrate binding and recognition (Muramatsu *et al.*, 2016 ▸). The great plasticity around the active site, involving the P2 helix, the adjacent loop and the P5 loop, has also been observed during ligand binding and makes the design of specific M^pro^ inhibitors a challenging task (Kneller *et al.*, 2020 ▸).

The P5 loop, the linker region between domain II and III consisting of residues 190 to 198, does not change its overall conformation at high pressure but exhibits shifts of its backbone (up to 0.52 Å for Ala193 C_α_). The temperature-dependent conformational heterogeneity between 100 K and 310 K previously described for the P5 loop involves lower flexibility at lower temperatures (Ebrahim *et al.*, 2022 ▸). The main-chain atom *B*-factor analysis reveals that high pressure also reduces the P5 loop flexibility, as the *B* factors are on average reduced by 3.2 Å^2^ in residues 191–198 compared with a reduction of only 0.8 Å^2^ on average for the whole protein chain at high pressure. The rotation of domain III gives rise to a regular checked pattern in the DDM, indicating single α-helices [Fig. 6 ▸(*a*)]. The largest pressure-induced movements in domain III are observed for residues 221–228, but drawing conclusions for this region is difficult due to the weak electron density around residue Arg222. Additionally important to note is that residues 74 and 222 are neighbors in the crystal structure, and the corresponding regions hence do not move completely independently.

Concluding, our findings for the effect of high pressure on SARS-CoV-2 M^pro^ agree well with the results of crystallographic studies systematically investigating the temperature-dependent conformational space of M^pro^ at temperatures ranging from 100 K to 310 K (Pearce *et al.*, 2017 ▸; Kneller *et al.*, 2020 ▸; Ebrahim *et al.*, 2022 ▸). These studies showed that M^pro^ exhibits a very high degree of flexibility in certain parts of the protein, including the P5 loop (residues 190–198), the P2 helix (residues 46–50), residues 68–76 of domain I, residues 92–97 of domain I and residues 218–227 of domain III. Thus, HP studies and studies at varying temperatures are complementary methods for investigating the local flexibility and conformational space of proteins.

## 4. Discussion

We have presented a new method for HP macromolecular crystallography experiments using helium gas as a pressurizing agent. Although helium is not supposed to interact with proteins and its solubility in water is rather low, data on the potential effects of helium on protein structures is scarce, to the best of our knowledge. Melnikov and co-workers reported that heavy noble gases affect the structure of membrane proteins by mimicking and altering protein–lipid interactions (Melnikov *et al.*, 2022 ▸). In general, noble gases like helium can interact with hydro­phobic surface areas of proteins via London dispersion forces, depending on the polarizability of the noble gas atoms (Quillin *et al.*, 2000 ▸; Prangé *et al.*, 2022 ▸). The polarizability, however, drops rapidly from heavier noble gases like xenon to lighter ones like helium (McNeill *et al.*, 2020 ▸). Therefore, selecting helium as the inert pressurizing agent allowed us to minimize potential interactions with the macromolecules.

Technologically, the HP cell is unique, as multiple im­mobilized protein crystals on a Kapton support can be measured at several pressure points and also after pressure release. This design allows complete X-ray diffraction data sets to be collected from low-symmetry crystals, as demonstrated for the 200 × 150 × 20 µm plate-like crystals of M^pro^ (space group *C*2), which appears challenging to study using conventional DACs with randomly oriented single crystals. The applicable pressure range of up to 120 MPa corresponds well with the ambient pressure in the Challenger Deep, the deepest region of the Earth’s oceans (10984 m), where life has been observed and is adapted to the environment (Todo *et al.*, 2005 ▸; Du *et al.*, 2021 ▸; Maguire *et al.*, 2024 ▸).

This experimental design ensures the immobilization and deliberate orientation of the protein crystals inside the cell, provides low background scattering due to the used low-*Z* materials when exposed to X-ray radiation with a photon energy of 26 keV, and avoids all issues that possibly accompany experiments applying liquid pressure media such as dissolving protein crystals or movement of crystals during X-ray exposure (Ahsbahs, 2004 ▸; Zegers *et al.*, 1994 ▸). The known position and orientation of the protein crystals on the Kapton support in the HP cell allow for rational sample-efficient collection of complete diffraction data sets at several pressure points, and even after pressure release, using exactly the same protein crystals in the same environment. We have demonstrated the general applicability of our method by determining the HP structures of five different proteins, including a membrane protein, at different pressures up to 120 MPa.

In agreement with previous studies, hydro­static high pressure enables a controlled and subtle compression of the crystal volume along all axes, allowing one to alter or enrich the conformational states. The solvent is nearly incompressible and the observed compression of the unit cells is mainly allowed by a reduction in void volume. The level of unit-cell compressibility strongly depends on the individual protein. Previously, pressure-induced collective displacements of protein atoms by up to around 1 Å at several hundred megapascals have been reported (Urayama *et al.*, 2002 ▸; Collins *et al.*, 2007 ▸; Barstow *et al.*, 2008 ▸). In a crystalline environment, crystal contacts can limit the protein’s conformational flexibility, and at increased hydro­static pressure surface side-chain orientations can potentially be altered. Crystal contact-dependent effects are however considered minor in comparison (Colloc’h *et al.*, 2023 ▸).

Generally, for all five proteins we analyzed, the unit-cell volumes decreased with increasing pressure, although the compressibilities varied considerably between them. Whereas the unit-cell volume of M^pro^ was only compressed by 0.15% at 110 MPa, the compression of AGE was about 20 times larger, being 2.8% already at 80 MPa. On a structural basis, this effect can be explained by compression of the central tunnel and more importantly of the AGE dimer interface. AGE catalyzes the reversible epimerization between *N*-acetyl-d-mannos­amine and *N*-acetyl-d-glucosamine, most probably via a deprotonation/reprotonation mechanism (Lee *et al.*, 2007 ▸; Lloyd *et al.*, 2021 ▸). In combination with an aldolase enzyme, AGE is used for biotechnological sialic acid synthesis. Due to the reduction in molar volume by the addition of a pyruvate molecule to *N*-acetyl-d-glucosamine to obtain sialic acid, this reaction is accelerated by higher pressures following Le Chatelier’s principle. Reich and co-workers showed that the position of the equilibrium, the selectivity and the reaction yield of the conversion of *N*-acetyl­mannosamine and pyruvate into sialic acid were improved significantly when pressure was applied to a continuous-flow fixed-bed reactor with immobil­ized enzymes (Reich *et al.*, 2022 ▸). However, the detailed reaction mechanism of AGE, the involved catalytic residues and the activity regulation via ATP-binding are still a matter of debate (Feng *et al.*, 2020 ▸; Klermund *et al.*, 2016 ▸; Lloyd *et al.*, 2021 ▸). The lack of this information hinders the development and study of pressure-adapted AGE mutants and their application in HP biotechnology. Further experiments are necessary to verify whether the observed structural changes correlate with the functional properties of *Pedobacter heparinus* AGE.

For RNase A we observed the smallest structural changes, and indeed this protein has been shown to be very pressure-resistant, preserving its structure even at a pressure above 0.67 GPa (Kurpiewska *et al.*, 2016 ▸). For the *Cereibacter sphaeroides* RC, to the best of our knowledge the first membrane protein studied with HP crystallography, the overall structural changes at 120 MPa are subtle as well. However, the ligand bound to the functionally relevant Q_B_ site was clearly stabilized by high pressure, highlighting the potential of HP experiments for a variety of ligand-binding studies. In a recent crystallographic study, Girard and co-workers showed that the binding of Zn^2+^-cyclen to a GTPase is strengthened at high pressure, driven by a pressure-dependent change of the preferred binding-site conformation (Girard *et al.*, 2024 ▸).

By adding a new dimension to the explorable free-energy landscape, these experiments are well suited to studying protein flexibility and dynamics by complementing other techniques such as multi-temperature experiments. From the point of view of applying pressure as a process parameter in enzyme-dependent biotechnological applications, this opens up benefits and new optimization possibilities, for example with regard to enzyme turnover, product yield and selectivity (Reich *et al.*, 2022 ▸; Eisenmenger & Reyes-De-Corcuera, 2009 ▸; Kumar *et al.*, 2023 ▸; Khan, 2025 ▸).

For the two pharmaceutically important proteins, DRAK2 and SARS-CoV-2 M^pro^, high pressure induces the largest structural rearrangements of the five studied proteins. Under pressure, the N- and C-lobes of DRAK2 move slightly towards each other, one small step on the trajectory of active-site cleft closure. The HP structure of M^pro^ clearly illustrates the structural flexibility around its active site. Knowledge of such conformational variability is essential for structure-based drug development, for the success of *in silico* compound screening methods, and as a basis for more accurate AI-assisted structure predictions including protein flexibility, thereby overcoming the single structure limit (Lane, 2023 ▸).

## Supplementary Material

Additional figures and tables. DOI: 10.1107/S2052252526008468/min5004sup1.pdf

PDB reference: RNase A at atmospheric pressure, 7q75

PDB reference: RNase A at 22 MPa, 7q76

PDB reference: RNase A at 50 MPa, 7q77

PDB reference: RNase A at 72 MPa, 7q78

PDB reference: RNase A at 100 MPa, 7q79

PDB reference: RNase A at 120 MPa, 7q7a

PDB reference: RNase A at atmospheric pressure after pressure release, 7q7b

PDB reference: DRAK2 at atmospheric pressure, 7q7c

PDB reference: DRAK2 at 110 MPa, 7q7d

PDB reference: DRAK2 at atmospheric pressure after pressure release, 7q7e

PDB reference: photosynthetic reaction center at atmospheric pressure, 7q7f

PDB reference: photosynthetic reaction center at 30 MPa, 7q7g

PDB reference: photosynthetic reaction center at 51 MPa, 7q7h

PDB reference: photosynthetic reaction center at 75 MPa, 7q7j

PDB reference: photosynthetic reaction center at 100 MPa, 7q7m

PDB reference: photosynthetic reaction center at 120 MPa, 7q7n

PDB reference: photosynthetic reaction center at atmospheric pressure after pressure release, 7q7o

PDB reference: *N*-acetyl­glucosamine 2-epimerase at atmospheric pressure, 8bry

PDB reference: *N*-acetyl­glucosamine 2-epimerase at 52 MPa, 8brz

PDB reference: *N*-acetyl­glucosamine 2-epimerase at 80 MPa, 8bs0

PDB reference: SARS-CoV-2 main protease at atmospheric pressure, 8bs1

PDB reference: SARS-CoV-2 main protease at 104 MPa, 8bs2

## Data Availability

All data are available in the main text or in the supplementary materials. All 22 determined protein structures have been deposited in the Protein Data Bank with the following PDB codes: 7q75 (RNase A at atmospheric pressure), 7q76 (RNase A at 22 MPa), 7q77 (RNase A at 50 MPa), 7q78 (RNase A at 72 MPa), 7q79 (RNase A at 100 MPa), 7q7a (RNase A at 120 MPa), 7q7b (RNase A at atmospheric pressure after pressure release), 7q7c (DRAK2 at atmospheric pressure), 7q7d (DRAK2 at 110 MPa), 7q7e (DRAK2 at atmospheric pressure after pressure release), 7q7f (photosynthetic reaction center at atmospheric pressure), 7q7g (photosynthetic reaction center at 30 MPa), 7q7h (photosynthetic reaction center at 51 MPa), 7q7j (photosynthetic reaction center at 75 MPa), 7q7m (photosynthetic reaction center at 100 MPa), 7q7n (photosynthetic reaction center at 120 MPa), 7q7o (photosynthetic reaction center at atmospheric pressure after pressure release), 8bry (*N*-acetyl­glucosamine 2-epi­merase at atmospheric pressure), 8brz (*N*-acetyl­glucosamine 2-epimerase at 52 MPa), 8bs0 (*N*-acetyl­glucosamine 2-epi­merase at 80 MPa), 8bs1 (SARS-CoV-2 main protease at atmospheric pressure) and 8bs2 (SARS-CoV-2 main protease at 104 MPa).
