# Supplementary material for: High-pressure macromolecular crystallography as a tool for studying protein flexibility
Source: IUCrJ. 2026 Oct 19;13(Pt 6):766–77. doi: 10.1107/S2052252526008468 (PMC13763890; doi:10.1107/S2052252526008468)
Supplement: Supplementary file 1 [file m-13-00766-sup1.pdf]

IUCrJ
Volume 13 (2026)
Supporting information for article:

High-pressure macromolecular crystallography as a tool for studying protein flexibility

Julia Lieske, Sofiane Saouane, Patrick Y. A. Reinke, Sven Falke, Wiebke Ewert, Jan Meyer, Tim Pakendorf, Anja Burkhardt, Eva Crosas, Johanna Hakanpää, Karolina Stachnik, Jochen Sieg, Matthias Rarey, Miriam Aßmann, Henning Zaun, Jürgen Kuballa, Georgii K. Selikhanov, Azat G. Gabdulkhakov, Henry N. Chapman, Sebastian Günther and Alke Meents

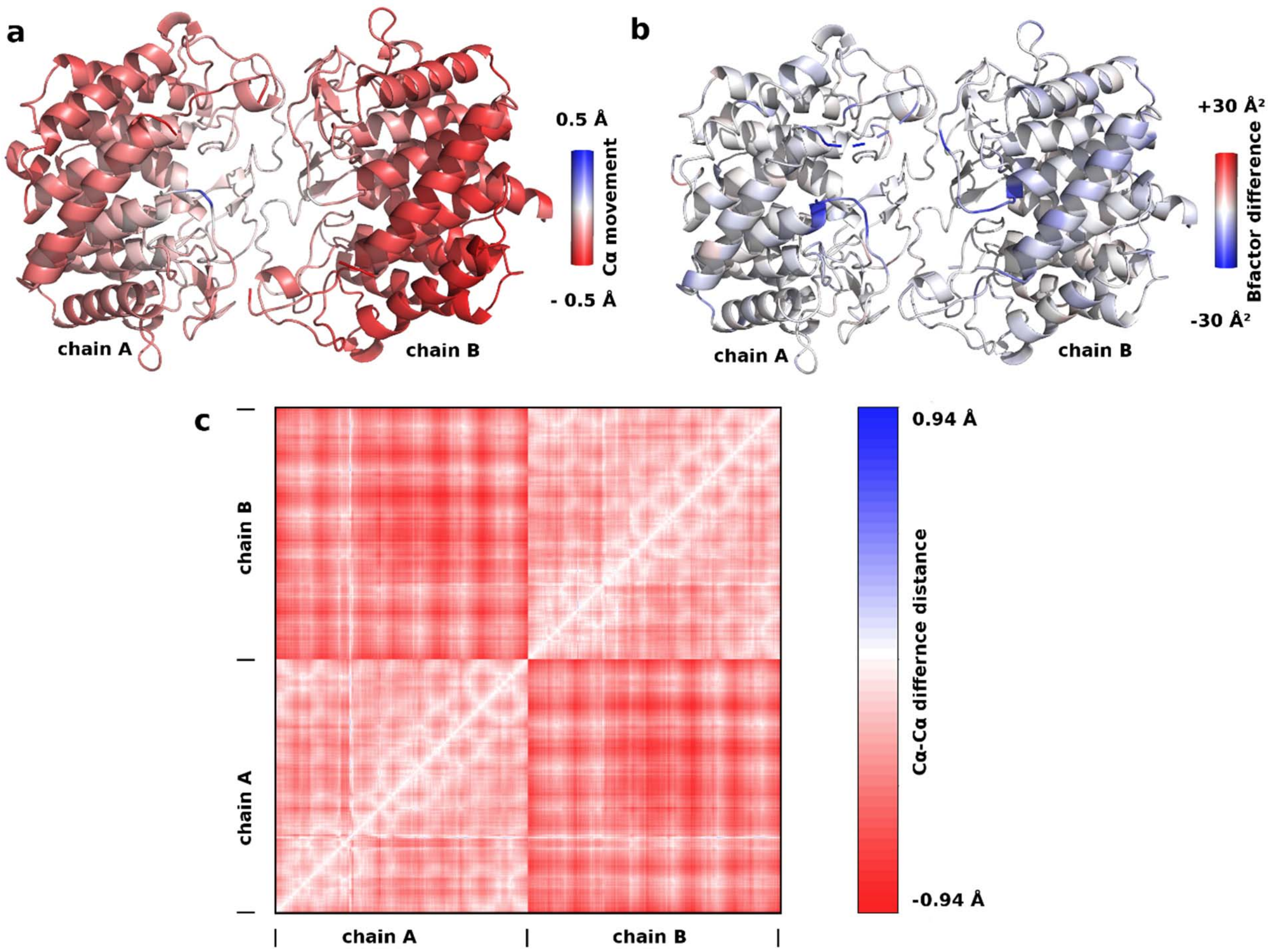

Figure S1 Results of the HP study of the *N*-acetylglucosamine 2-epimerase (AGE). (**a**) C_α movement in respect to the dimer center of mass between the structures at atmospheric pressure and 80 MPa helium pressure plotted onto the dimer structure. (**b**) Overall B-factor differences between the structures at atmospheric pressure and 80 MPa helium pressure plotted onto the dimer structure. The AGE structure exhibits lower B factors (on average by -1.34 $Å^2$ and -1.87 $Å^2$ for chain A and B, respectively) and hence less flexibility at HP. (**c**) Difference distance matrix between the AGE structures at atmospheric pressure and at 80 MPa.

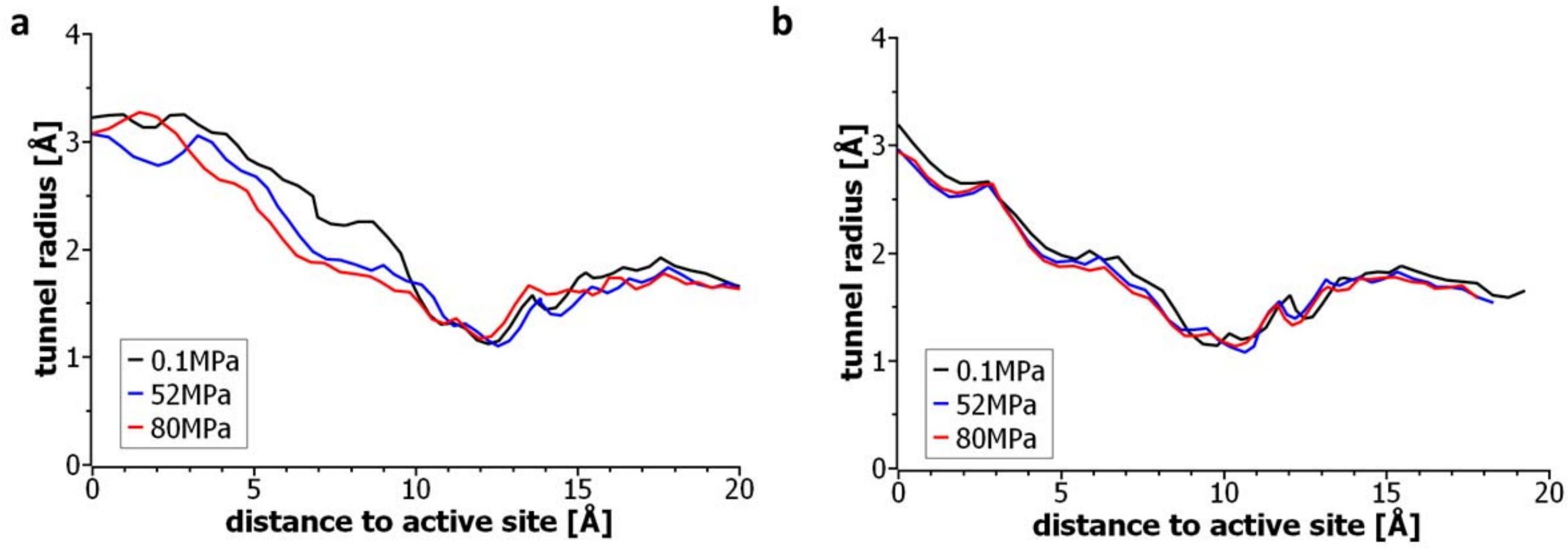

Figure S2 Plot of the radii of the central tunnels of the AGE (α/α)$_6$-barrel monomers A (**a**) and B (**b**) against the distance to the corresponding active site at different pressure levels. High helium pressure leads to compression of the central tunnels but without changing the minimum radius at the tunnel bottleneck. This effect is more pronounced in monomer A than in B.

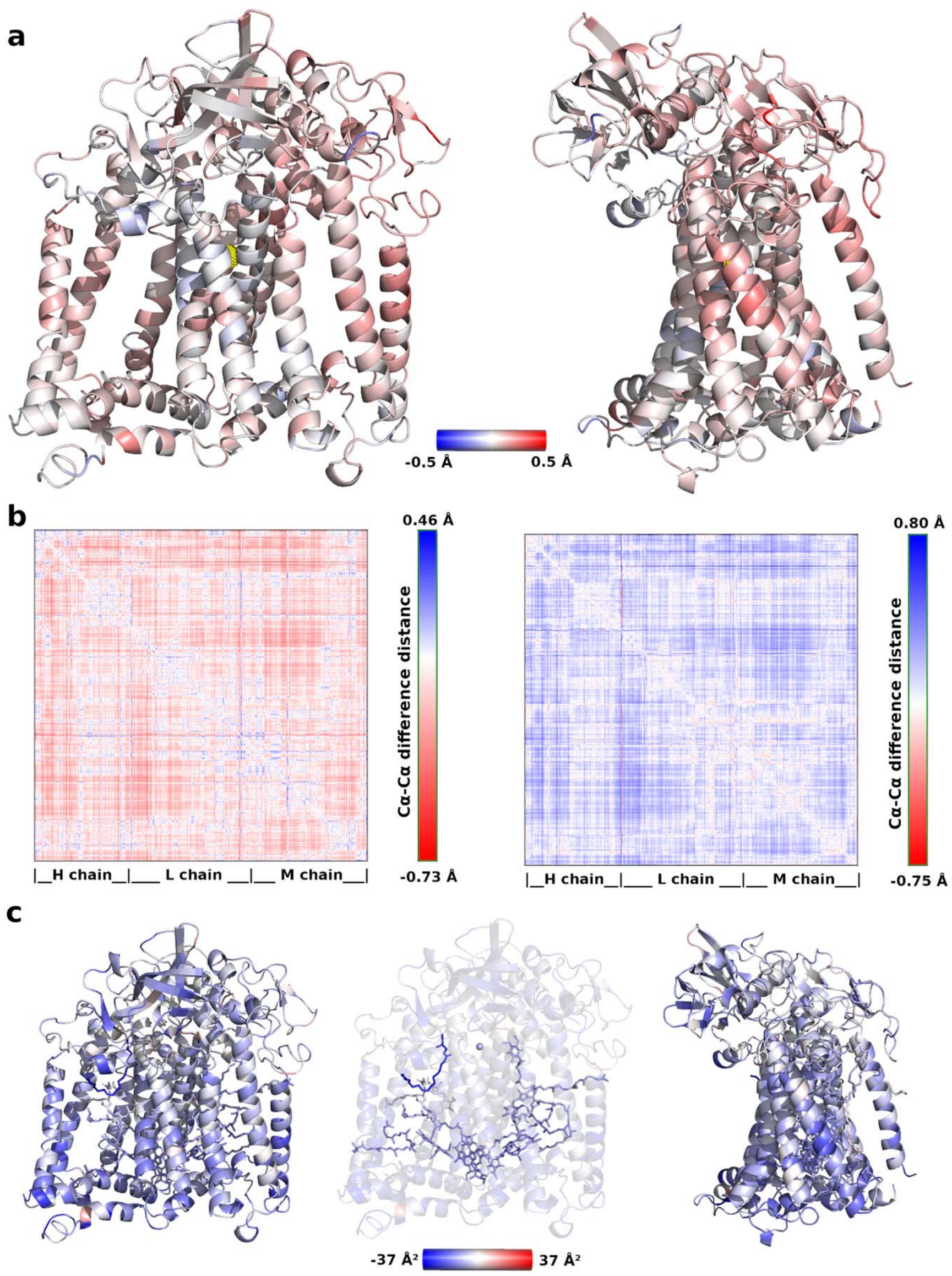

Figure S3 Comparison of the reaction center structures at 120 MPa and at 0.1 MPa. (**a**) Depiction of the pressure-induced C_α movements in respect to the center of mass of the protein (*yellow sphere*) in front and side view. It shows that the RC is slightly compressed under HP. Positive values (*red*) indicate that atoms are closer to the center when pressure is applied, negative values (*blue*) that atoms are further away under pressure. Scales are adapted to the maximal observed differences. (**b**) Difference distance matrices of the RC structures at 0.1 MPa and 120 MPa, chains ordered as HLM. During pressurization (left) overall difference distances show a slight compression of the protein complex, whereas in reverse decompression is observed upon pressure release (*right*). Patterns in the DDM indicate areas of concerted movements of adjacent residues. (**c**) Absolute B factor differences between the structures at 120 MPa and 0.1 MPa plotted onto the RC structure, in front view (*left*), details of the ten bound cofactors (*middle*) and in side view (*right*). At 120 MPa, B factors are decreased on average by 8.4 $Å^2$ for all atoms and by 12.5 $Å^2$ for all atoms of the ten cofactors. Red and blue colors indicate a B factor increase and decrease, respectively, at HP. Scales are adapted to the maximal observed differences.

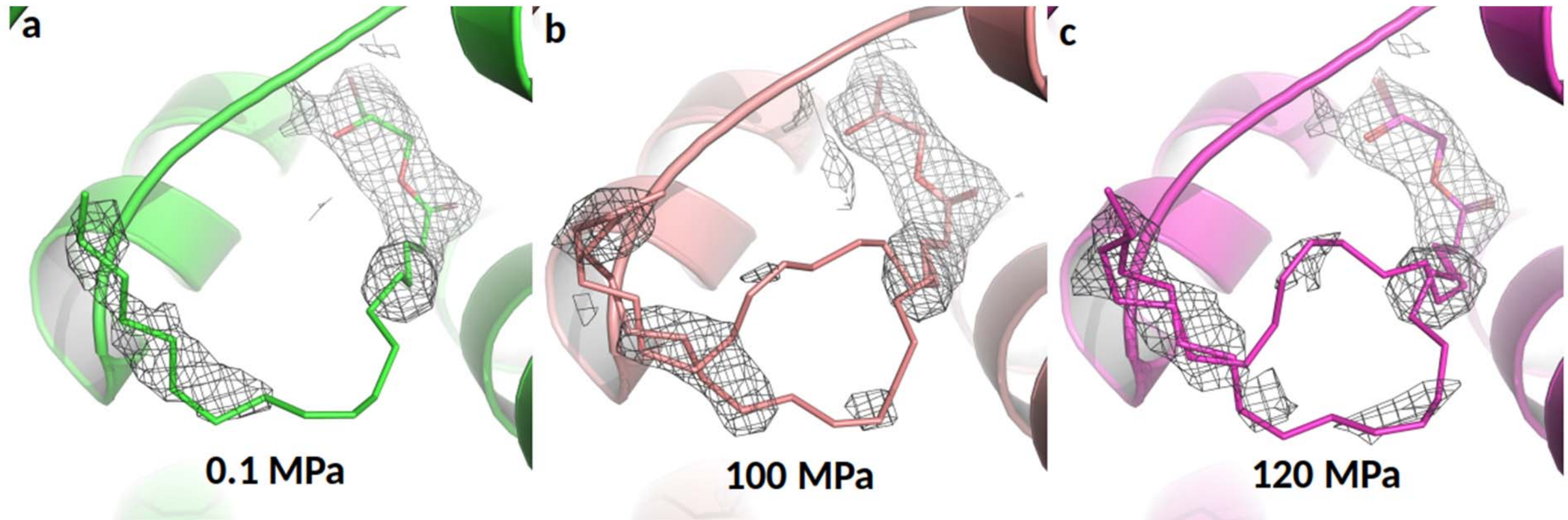

Figure S4 Comparison of monoolein binding to the Q_B site of the RC at atmospheric pressure (**a**), at 100 MPa (**b**) and at 120 MPa (**c**). Depicted are the $2mF_o$-DF_c electron density maps at 0.75 σ level as grey meshes, the monoolein in sticks and the protein in cartoon representation.

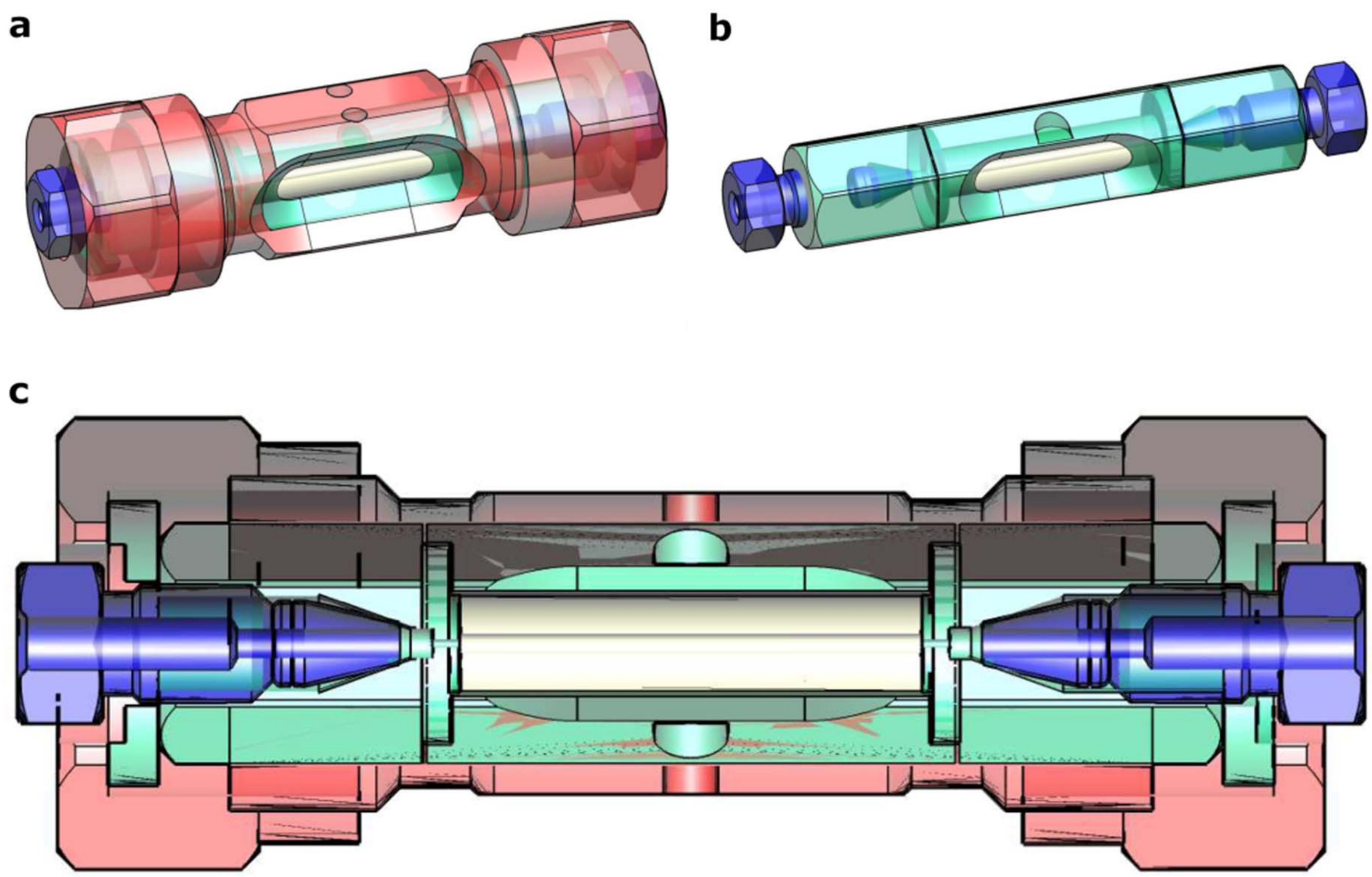

Figure S5 Schematic drawings of the new HP cell. Close-up views of the fully assembled HP cell (**a**), the inner parts (**b**) and a cut through (**c**). The central single-crystalline sapphire capillary (*yellow*, provider: GWI – Grazyna Walawski Industriesaphire, Birkenstr. 5, D-91287 Plech) has a length of 15 mm with an inner and outer diameter of 0.7 mm and 3 mm, respectively. It is embedded into a stainless-steel cage (*green*) providing a window with an effectively usable length of 8 mm and an opening angle of 90 degrees allowing for shadowless 45-degree rotation data collection. Two customized fittings at both sides of the cage (*green*) provide the connection for commercially available 1/32" standard nuts and ferrules (*blue*). All inner parts are held together by a surrounding jack and two large screws at both ends that are used to tighten the cell (*red*).

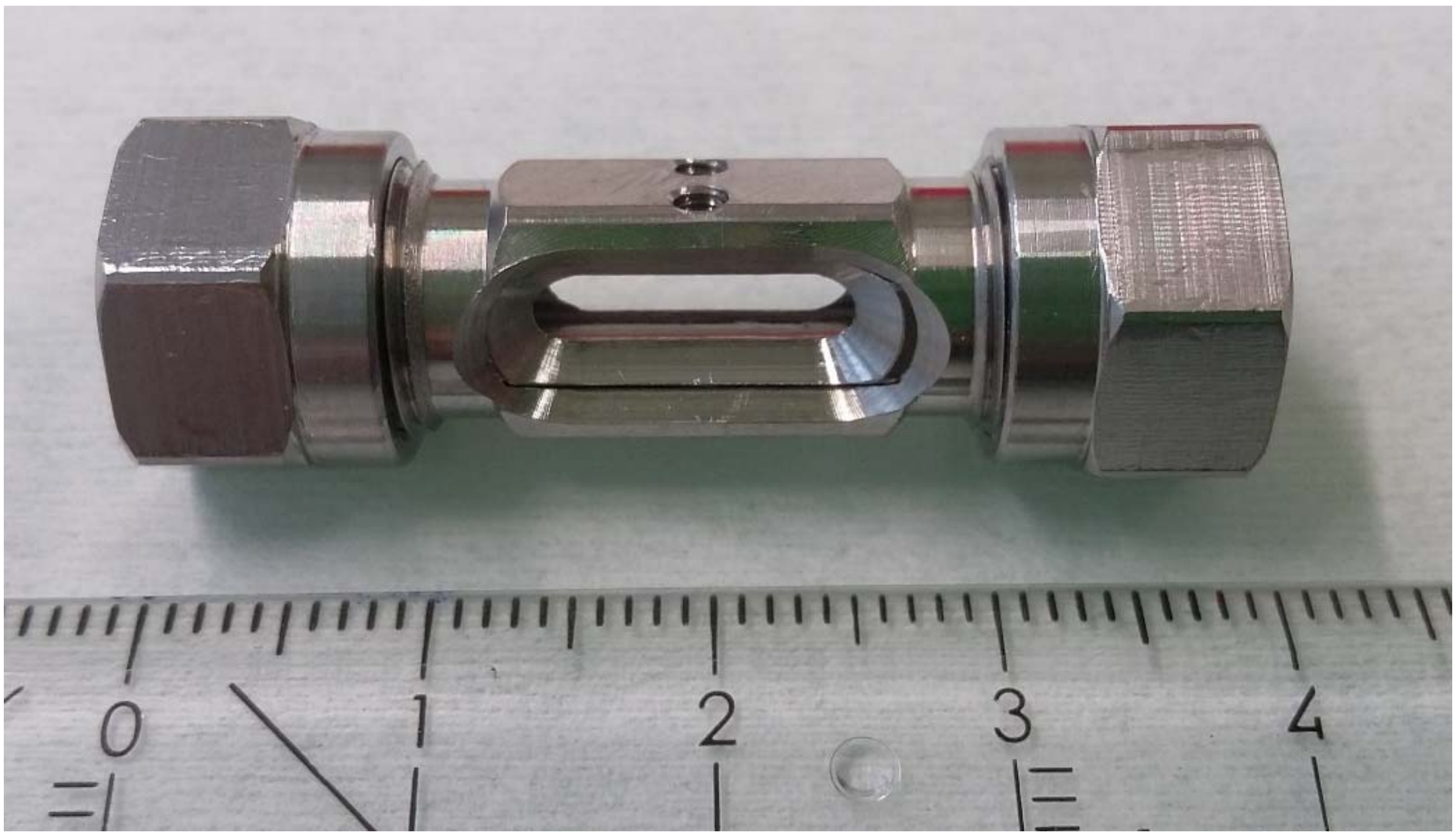

Figure S6 Picture of the HP cell with cm scale.

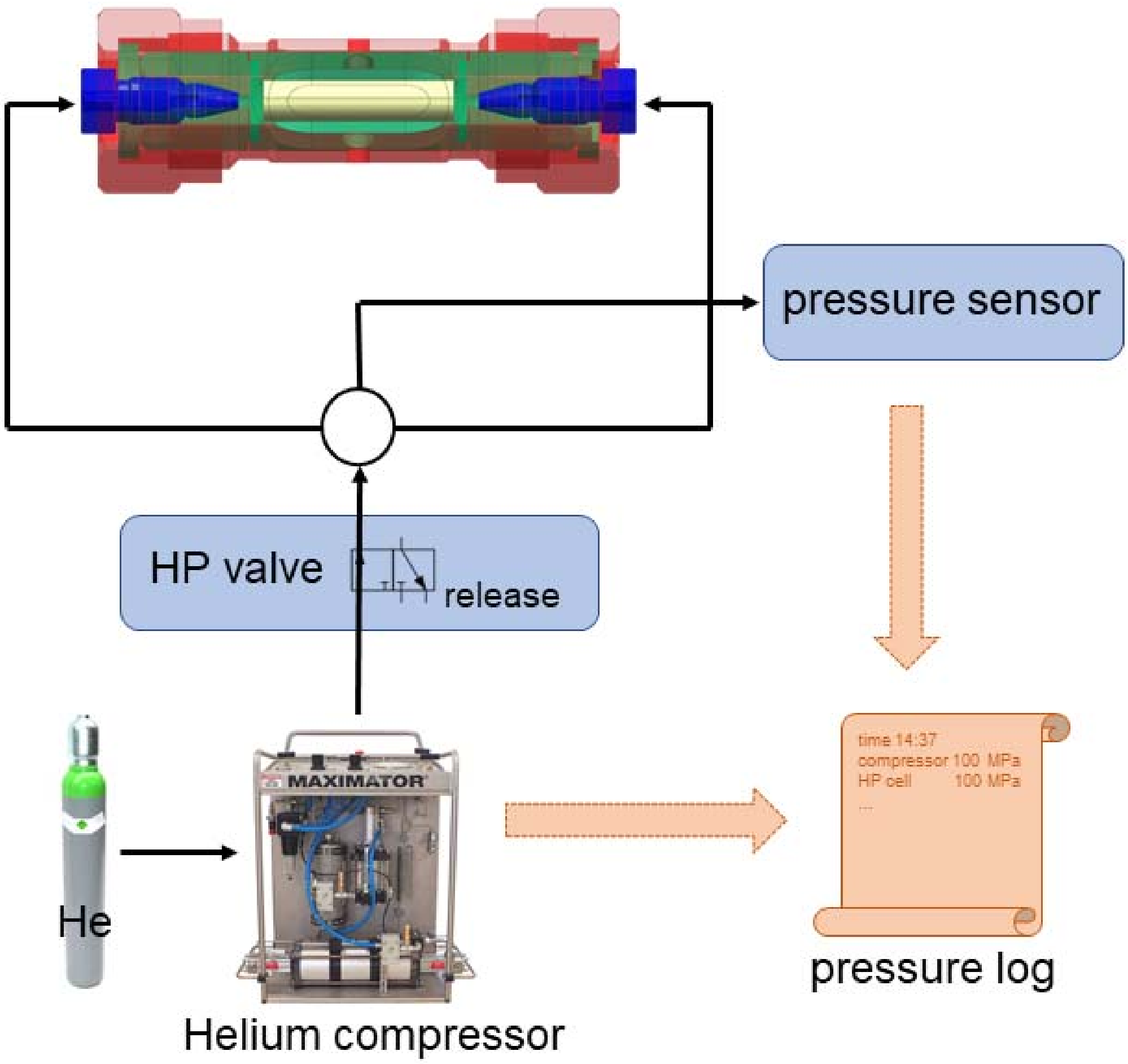

Figure S7 Schematic picture of the HP setup for macromolecular crystallography at DESY PETRA III P11 beamline.

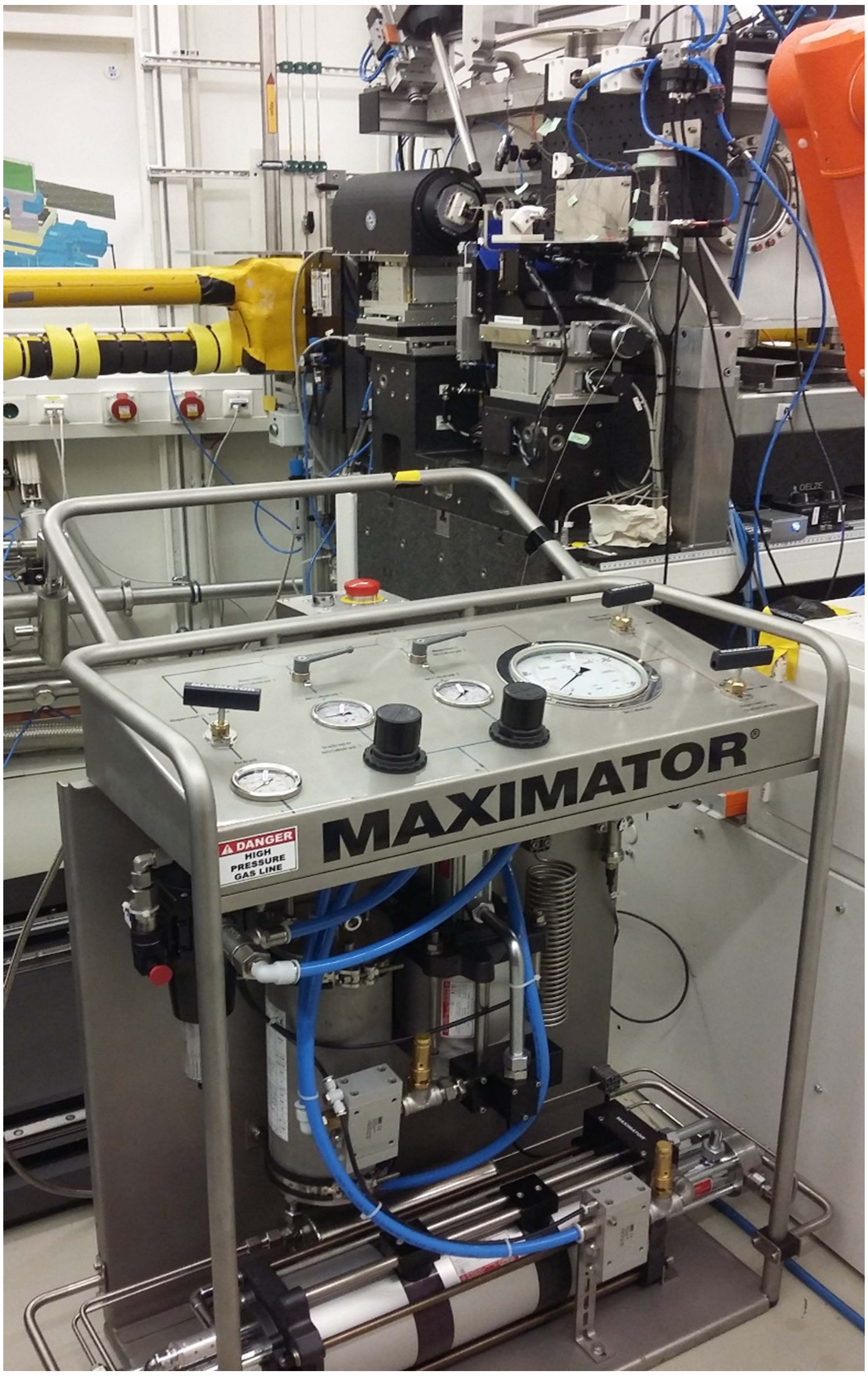

Figure S8 Picture of the experimental setup at the DESY PETRA III P11 beamline with the double-stage helium compressor in front.

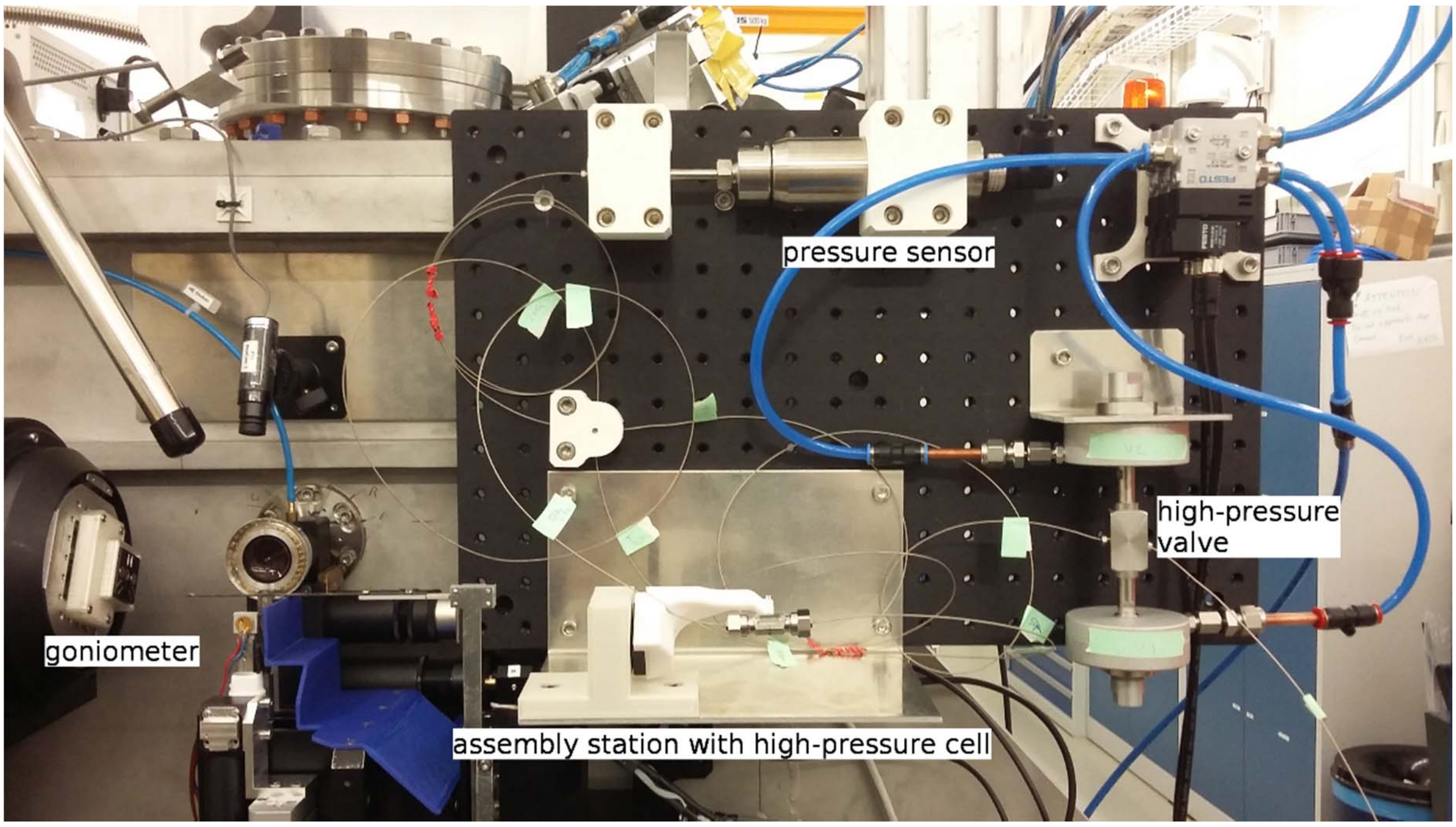

Figure S9 Details of the experimental setup at the DESY PETRA III P11 beamline showing the cell assembly station, the HP valves and the pressure sensor mounted on a breadboard.

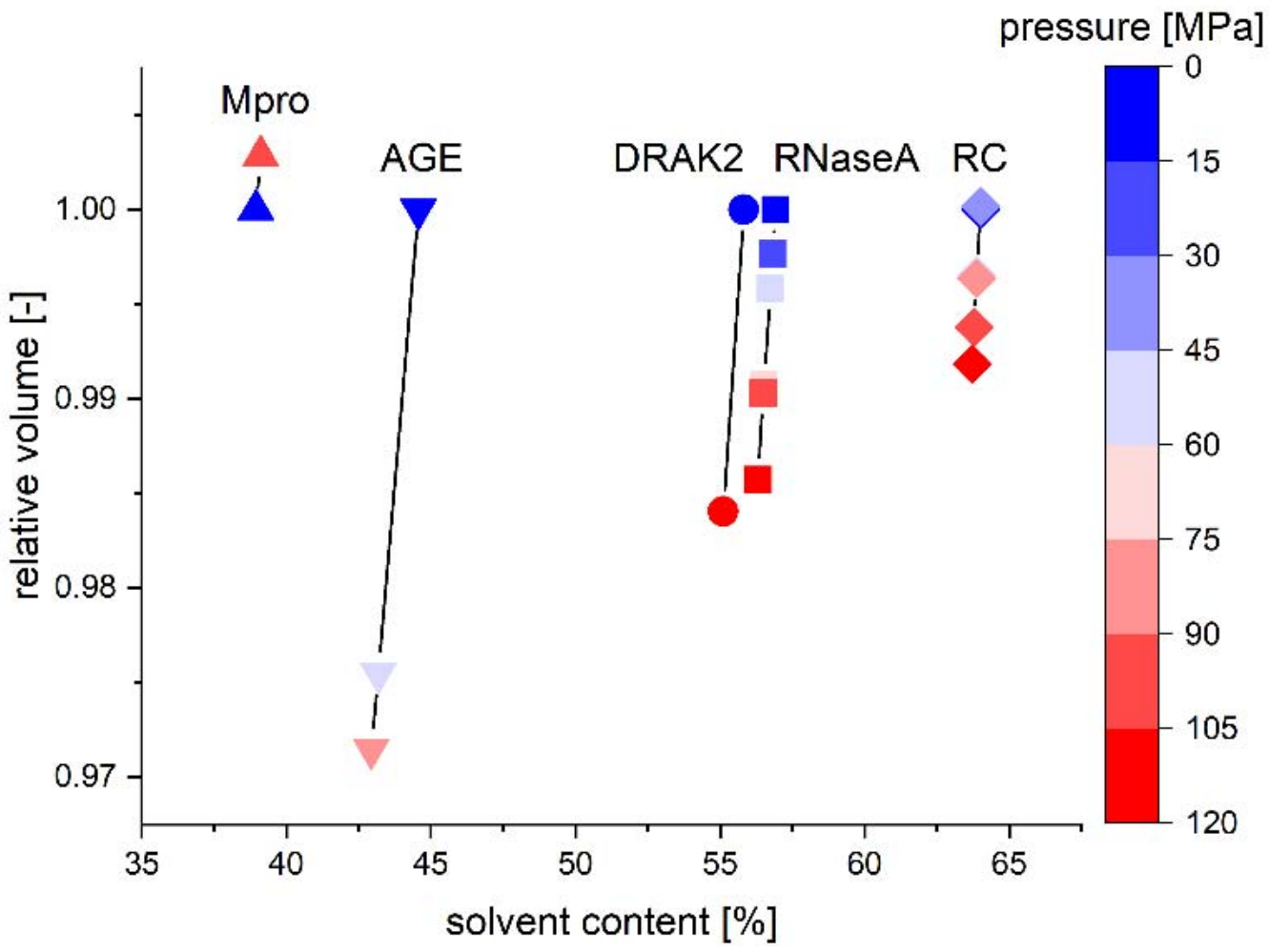

Figure S10 Volume compression dependence on solvent content of crystals.

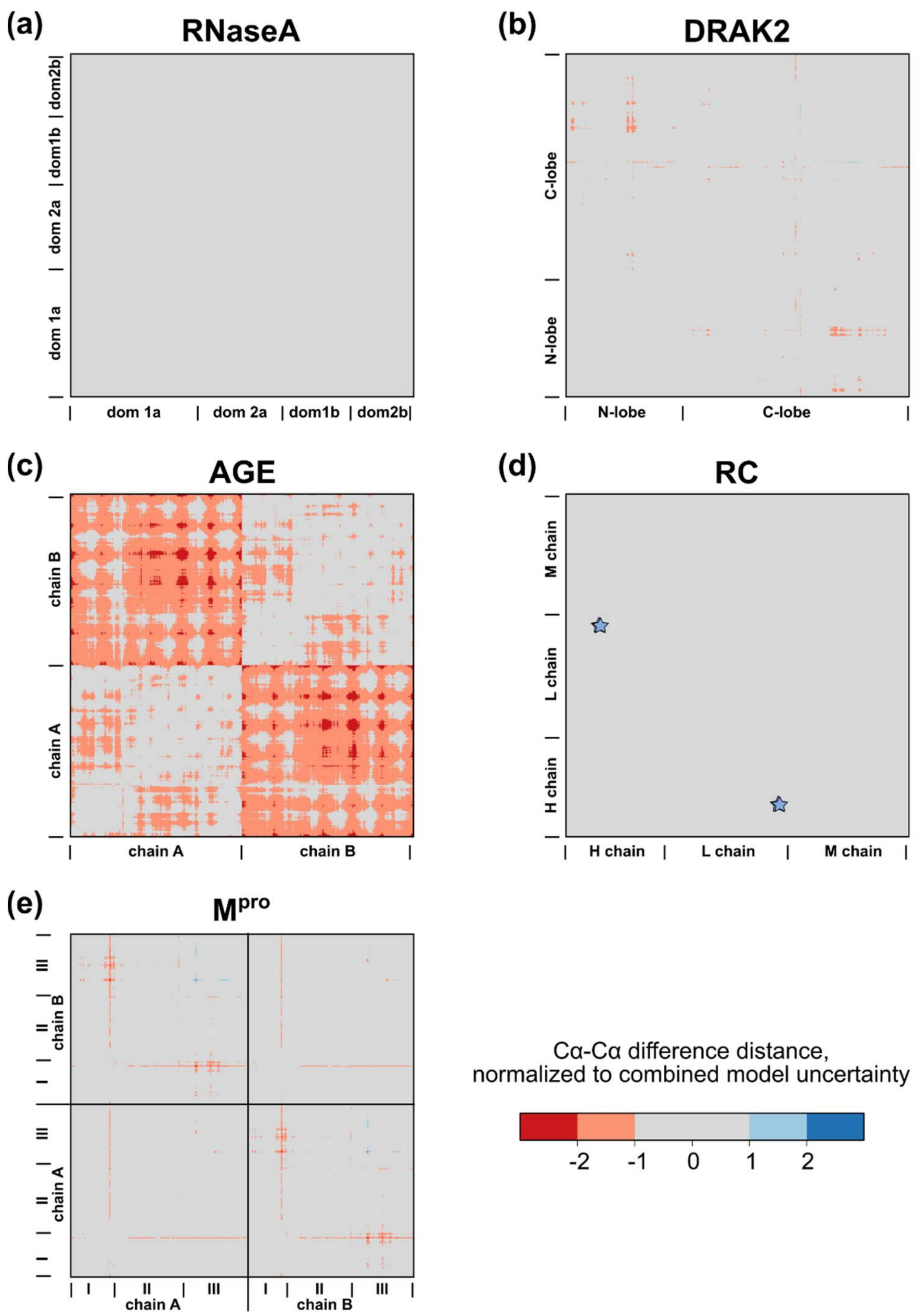

Figure S11 Cα-Cα difference distance matrices identify regions with pressure-induced movements. Depiction similar to Figure 4 and 6a with distances normalized to the combined coordinate uncertainty of the compared structures (highest pressure with ambient pressure structure). (**a**) RNaseA, 0.445 Å; (**b**) DRAK2, 0.688 Å; (**c**) AGE, 0.332 Å; (**d**) RC, 0.661 Å; (**e**) M^{pro}, 0.587 Å. No movement above the coordinate uncertainty was observed for RNaseA, and only two for RC, marked by a star.

Table S1 Data collection and refinement statistics for the HP **RNase A** structures.

**Applied Pressure**	**0.1 MPa**	**22 MPa**	**50 MPa**	**72 MPa**	**100 MPa**	**120 MPa**	**0.1 MPa after pressure release**
**PDB ID**	**7Q75**	**7Q76**	**7Q77**	**7Q78**	**7Q79**	**7Q7A**	**7Q7B**
No. of merged datasets	5	4	3	4	3	3	2
No. of used HP cell loadings	2	1	1	1	1	1	1
Space group	P 3_2 2 1	P 3_2 2 1	P 3_2 2 1	P 3_2 2 1	P 3_2 2 1	P 3_2 2 1	P 3_2 2 1
Cell dimensions							
a, b, c (Å)	64.52, 64.52, 65.14	64.42, 64.42, 65.19	64.49, 64.49, 64.93	64.28, 64.28, 65.02	64.40, 64.40, 64.75	64.28, 64.28, 64.69	64.67, 64.67, 65.22
α, β, γ (°)	90, 90, 120	90, 90, 120	90, 90, 120	90, 90, 120	90, 90, 120	90, 90, 120	90, 90, 120
Resolution range (Å)	28.14 - 1.64 (1.699 - 1.64)	25.65 - 1.48 (1.533 - 1.48)	27.92 - 1.6 (1.657 - 1.6)	25.59 - 1.52 (1.574 - 1.52)	27.89 - 1.55 (1.605 - 1.55)	27.97 - 1.56 (1.616 - 1.56)	28 - 1.78 (1.844 - 1.78)
Total reflections	218938 (21279)	221475 (20763)	141084 (13617)	214583 (21150)	155140 (14923)	150454 (14499)	70190 (6311)
Unique reflections	19611 (1936)	26313 (2593)	20806 (2069)	24342 (2416)	22676 (2245)	22159 (2212)	15243 (1502)
Multiplicity	11.2 (11.0)	8.4 (8.0)	6.8 (6.6)	8.8 (8.8)	6.8 (6.6)	6.8 (6.6)	4.6 (4.2)
Completeness (%)	99.79 (99.38)	99.17 (98.67)	98.76 (99.23)	99.93 (99.92)	98.72 (99.60)	98.66 (99.33)	98.16 (98.55)
Mean I/sigma(I)	7.76 (1.02)	7.44 (0.99)	5.83 (1.08)	8.24 (1.01)	6.11 (1.01)	6.7 (1.0)	7.38 (0.96)
Wilson B-factor	23.05	21.28	22.00	19.94	21.53	20.96	29.19
R-merge	0.1685 (2.139)	0.14 (2.128)	0.1792 (2.115)	0.1495 (2.602)	0.1619 (1.905)	0.144 (1.913)	0.1107 (1.512)

R-meas	0.1764 (2.244)	0.1487 (2.276)	0.1937 (2.303)	0.1585 (2.762)	0.1746 (2.065)	0.155 (2.076)	0.1245 (1.727)
R-pim	0.0515 (0.6683)	0.0485 (0.7747)	0.0720 (0.8893)	0.0519 (0.917)	0.0642 (0.7833)	0.0570 (0.795)	0.0559 (0.8206)
$CC_{1/2}$	0.995 (0.334)	0.991 (0.304)	0.987 (0.334)	0.995 (0.327)	0.988 (0.306)	0.993 (0.374)	0.993 (0.311)
Reflections used in refinement	19596 (1931)	26303 (2591)	20788 (2059)	24338 (2415)	22672 (2245)	22153 (2211)	15234 (1500)
Reflections used for R-free	1535 (155)	2075 (210)	1633 (169)	1916 (194)	1779 (170)	1742 (175)	1191 (120)
R-work	0.1548 (0.3359)	0.1494 (0.2908)	0.1538 (0.2904)	0.1533 (0.3117)	0.1558 (0.3181)	0.1548 (0.3005)	0.1489 (0.3419)
R-free	0.1883 (0.3724)	0.1793 (0.3095)	0.1877 (0.2948)	0.1823 (0.3506)	0.1863 (0.3607)	0.1896 (0.3550)	0.1842 (0.3733)
Number of non-hydrogen atoms	1136	1132	1135	1124	1141	1157	1122
macromolecules	1041	1041	1041	1030	1047	1047	1041
ligands	14	14	14	14	14	14	14
solvent	81	77	80	80	80	96	67
Protein residues	124	124	124	124	124	124	124
RMS(bonds)	0.009	0.014	0.009	0.015	0.010	0.008	0.011
RMS(angles)	1.33	1.53	1.31	1.52	1.30	1.23	1.32
Ramachandran favored (%)	97.54	95.90	97.54	96.72	97.54	96.72	96.72
Ramachandran allowed (%)	2.46	4.10	2.46	3.28	2.46	3.28	3.28
Ramachandran outliers (%)	0.00	0.00	0.00	0.00	0.00	0.00	0.00
Rotamer outliers (%)	2.75	0.92	2.75	2.75	3.67	1.83	3.67
Clashscore	5.25	4.77	6.20	5.31	5.20	4.26	4.77
Average B-factor	32.26	29.96	31.09	28.15	31.46	30.12	38.91
macromolecules	31.07	28.95	29.92	27.09	30.34	28.86	37.87

ligands	64.84	57.90	62.64	55.19	65.11	60.19	79.94
solvent	42.03	38.55	40.69	37.13	40.22	39.40	46.44
Coordinate error (Å)	0.243	0.179	0.179	0.202	0.211	0.201	0.243
Number of TLS groups	10	7	10	10	10	10	10

Statistics for the highest-resolution shell are shown in parentheses.

Table S2 Data collection and refinement statistics for the HP ***N*-acetylglucosamine 2-epimerase** structures.

**Applied Pressure**	**0.1 MPa**	**52 MPa**	**80 MPa**
**PDB ID**	**8BRY**	**8BRZ**	**8BS0**
No. of merged datasets	1	10	11
No. of used HP cell loadings	measured in loop	2	2
Resolution range(Å)	17.62 - 1.5 (1.554 - 1.5)	41.28 - 1.7 (1.761 - 1.7)	39.79 - 1.7 (1.761 - 1.7)
Space group	$P\ 1\ 2_1\ 1$	$P\ 1\ 2_1\ 1$	$P\ 1\ 2_1\ 1$
Cell dimensions			
a, b, c (Å)	56.945, 94.626, 82.465	56.41, 94.08, 81.71	56.35, 93.98, 81.52
α, β, γ (°)	90, 102.53, 90	90, 102.63, 90	90, 102.55, 90
Total reflections	922945 (89971)	703262 (48018)	674075 (31386)
Unique reflections	135935 (13529)	88642 (8015)	81812 (5880)
Multiplicity	6.8 (6.7)	7.9 (6.0)	8.2 (5.3)
Completeness (%)	99.67 (99.29)	96.35 (82.55)	89.75 (64.51)
Mean I/sigma(I)	5.83 (0.89)	5.64 (0.95)	5.85 (0.99)
Wilson B-factor	20.53	17.71	20.51
R-merge	0.176 (1.755)	0.2875 (1.751)	0.2657 (1.36)
R-meas	0.1914 (1.905)	0.3072 (1.892)	0.2824 (1.469)
R-pim	0.0744 (0.7352)	0.1042 (0.6563)	0.0907 (0.5262)
$CC_{1/2}$	0.994 (0.495)	0.983 (0.242)	0.985 (0.434)

CC*	0.998 (0.814)	0.996 (0.624)	0.996 (0.778)
Reflections used in refinement	135727 (13451)	88072 (7519)	81713 (5852)
Reflections used for R-free	1305 (130)	1524 (130)	1749 (126)
R-work	0.1537 (0.2957)	0.1574 (0.2936)	0.1559 (0.2641)
R-free	0.1631 (0.2920)	0.1828 (0.3545)	0.1803 (0.2827)
No. of non-hydrogen atoms	7161	7120	7006
macromolecules	6677	6634	6573
ligands	12	12	12
solvent	472	474	421
Protein residues	774	772	772
RMS(bonds)	0.008	0.004	0.008
RMS(angles)	1.04	0.69	0.79
Ramachandran favored (%)	97.65	97.25	97.64
Ramachandran allowed (%)	2.35	2.62	2.23
Ramachandran outliers (%)	0.00	0.13	0.13
Rotamer outliers (%)	0.3	0.46	0.46
Clashscore	1.91	1.31	1.79
Average B-factor	29.27	24.40	27.41
macromolecules	28.48	23.80	26.94
ligands	46.44	33.48	36.17
solvent	40.07	32.63	34.39
Coordinate error (Å)	0.174	0.180	0.158
No. of TLS groups	6	11	10

Statistics for the highest-resolution shell are shown in parentheses.

Table S3 Data collection and refinement statistics for the HP **photosynthetic reaction center (RC)** structures.

**Applied Pressure**	**0.1 MPa**	**30 MPa**	**51 MPa**	**75 MPa**	**100 MPa**	**120 MPa**	**0.1 MPa after pressure release**
**PDB ID**	**7Q7F**	**7Q7G**	**7Q7H**	**7Q7J**	**7Q7M**	**7Q7N**	**7Q7O**
No. of merged datasets	6	5	4	5	9	3	10
No. of used HP cell loadings	3	1	1	1	2	1	1
Resolution range(Å)	45.97 - 2.75 (2.848 - 2.75)	47.05 - 2.69 (2.786 - 2.69)	46.99 - 2.49 (2.579 - 2.49)	45.94 - 2.69 (2.786 - 2.69)	46.95 - 2.55 (2.641 - 2.55)	46.91 - 2.87 (2.973 - 2.87)	45.96 - 2.65 (2.745 - 2.65)
Space group	$P\ 4_2\ 2_1\ 2$	$P\ 4_2\ 2_1\ 2$	$P\ 4_2\ 2_1\ 2$	$P\ 4_2\ 2_1\ 2$	$P\ 4_2\ 2_1\ 2$	$P\ 4_2\ 2_1\ 2$	$P\ 4_2\ 2_1\ 2$
Cell dimensions							
a, b, c (Å)	102.50, 102.50, 237.84	102.44, 102.44, 238.16	102.29, 102.29, 237.99	102.30, 102.30, 237.91	102.21, 102.21, 237.71	102.13, 102.13, 237.61	102.52, 102.52, 237.68
α, β, γ (°)	90, 90, 90	90, 90, 90	90, 90, 90	90, 90, 90	90, 90, 90	90, 90, 90	90, 90, 90
Total reflections	362813 (32978)	387325 (33731)	376334 (32022)	383829 (33487)	722118 (66583)	190631 (18940)	646109 (57346)
Unique reflections	33826 (3326)	35870 (3499)	44663 (4379)	35764 (3422)	41962 (4104)	28804 (2803)	37727 (3691)
Multiplicity	10.7 (9.9)	10.8 (9.6)	8.4 (7.3)	10.7 (9.8)	17.2 (16.2)	6.6 (6.8)	17.1 (15.5)
Completeness (%)	99.77 (99.61)	99.16 (98.64)	98.85 (99.18)	99.28 (97.37)	99.85 (99.95)	96.97 (96.07)	99.86 (99.95)
Mean I/sigma(I)	5.13 (1.02)	6.01 (1.04)	5.86 (1.01)	5.04 (0.99)	5.93 (1.00)	3.39 (0.98)	6.00 (0.96)
Wilson B-factor	49.57	44.54	40.43	42.04	37.37	45.83	46.12

R-merge	0.4138 (2.351)	0.3716 (2.099)	0.3061 (2.105)	0.4511 (2.294)	0.5095 (2.931)	0.4805 (2.012)	0.4758 (3.133)
R-meas	0.4345 (2.475)	0.3903 (2.217)	0.3262 (2.266)	0.4736 (2.422)	0.5252 (3.027)	0.5178 (2.163)	0.4904 (3.238)
R-pim	0.1283 (0.7511)	0.1155 (0.6855)	0.1097 (0.8181)	0.1388 (0.7479)	0.1242 (0.7395)	0.1868 (0.7717)	0.1157 (0.7977)
$CC_{1/2}$	0.976 (0.355)	0.978 (0.32)	0.984 (0.347)	0.968 (0.347)	0.979 (0.375)	0.935 (0.367)	0.982 (0.329)
CC*	0.994 (0.724)	0.994 (0.696)	0.996 (0.717)	0.992 (0.718)	0.995 (0.738)	0.983 (0.733)	0.995 (0.703)
Reflections used in refinement	33810 (3320)	35830 (3473)	44643 (4375)	35739 (3410)	41947 (4103)	28770 (2784)	37712 (3690)
Reflections used for R-free	1358 (134)	1440 (141)	1794 (176)	1436 (137)	1686 (164)	1157 (112)	1515 (149)
R-work	0.1788 (0.2828)	0.1750 (0.2868)	0.1766 (0.2881)	0.1788 (0.2871)	0.1831 (0.2904)	0.1892 (0.2822)	0.1760 (0.2934)
R-free	0.2239 (0.3194)	0.2237 (0.3143)	0.2113 (0.3006)	0.2294 (0.3533)	0.2225 (0.3297)	0.2277 (0.3600)	0.2161 (0.3274)
No. of non-hydrogen atoms	7382	7332	7443	7390	7475	7387	7365
macromolecules	6553	6550	6554	6550	6580	6545	6538
ligands	674	637	689	674	700	699	674
solvent	155	145	200	166	195	143	153
Protein residues	821	821	821	821	821	820	821
RMS(bonds)	0.009	0.009	0.009	0.009	0.009	0.009	0.009
RMS(angles)	1.10	1.15	1.12	1.15	1.12	1.11	1.12
Ramachandran favored (%)	97.91	98.04	97.55	97.55	98.04	97.54	98.65
Ramachandran allowed (%)	2.09	1.96	2.33	2.21	1.96	2.33	1.23

Ramachandran outliers (%)	0.00	0.00	0.12	0.25	0.00	0.12	0.12
Rotamer outliers (%)	1.69	1.69	0.92	1.54	1.69	1.38	1.84
Clashscore	2.27	3.05	2.40	2.69	2.46	2.88	2.14
Average B-factor	50.59	46.65	45.89	43.33	44.99	42.91	49.11
macromolecules	50.44	46.41	45.32	42.94	44.03	42.76	48.86
ligands	54.61	51.34	52.53	48.42	54.42	46.64	53.26
solvent	39.62	36.90	41.64	37.86	43.79	31.47	41.43
Coordinate error (Å)	0.328	0.305	0.267	0.354	0.276	0.333	0.329
No. of TLS groups	17	17	16	17	17	17	17

Statistics for the highest-resolution shell are shown in parentheses

Table S4 Data collection and refinement statistics for the HP **DRAK2** and HP **SARS-CoV-2 M^{pro}** structures.

**Applied Pressure**	**DRAK2** **0.1 MPa**	**DRAK2** **110 MPa**	**DRAK2** **0.1 MPa after pressure release**	**M^{pro}** **0.1 MPa**	**M^{pro}** **104 MPa**
**PDB ID**	**7Q7C**	**7Q7D**	**7Q7E**	**8BS1**	**8BS2**
No. of merged datasets	35	9	4	5	16
No. of used HP cell loadings	Measured on silicon chip	1	1	measured in loop	4
Resolution range (Å)	47.98 - 2.85 (2.952 - 2.85)	39.46 - 2.6 (2.693 - 2.6)	48.12 - 2.85 (2.952 - 2.85)	26.18 - 2.05 (2.123 - 2.05)	25.95 - 2.35 (2.434 - 2.35)
Space group	P 4_1 2 2	P 4_1 2 2	P 4_1 2 2	C 1 2 1	C 1 2 1
Cell dimensions					
a, b, c (Å)	84.44, 84.44, 116.61	83.89, 83.89, 116.26	84.24, 84.24, 117.26	114.17, 54.17, 45.09	113.58, 54.5, 45.05
α, β, γ (°)	90, 90, 90	90, 90, 90	90, 90, 90	90, 102.041, 90	90, 101.246, 90
Total reflections	79792 (5372)	334357 (28421)	198561 (19647)	258134 (22335)	113742 (6418)
Unique reflections	10282 (982)	12947 (1181)	9542 (930)	17033 (1685)	11107 (991)
Multiplicity	7.8 (5.5)	25.8 (24.1)	20.8 (21.1)	15.2 (13.3)	10.2 (6.5)
Completeness (%)	98.91 (97.31)	96.68 (89.20)	91.75 (92.90)	99.89 (99.94)	97.60 (86.87)
Mean I/sigma(I)	5.93 (0.99)	13.03 (1.14)	9.44 (1.10)	6.73 (1.01)	8.40 (1.19)
Wilson B-factor	92.37	62.73	69.36	42.93	41.23
R-merge	0.2012 (1.085)	0.2131 (2.875)	0.3501 (3.753)	0.2655 (2.182)	0.2854 (1.823)
R-meas	0.2153 (1.195)	0.2176 (2.937)	0.3588 (3.845)	0.2743 (2.27)	0.3003 (1.963)

R-pim	0.0741 (0.4863)	0.0418 (0.5836)	0.0760 (0.8122)	0.0687 (0.6218)	0.0883 (0.6839)
$CC_{1/2}$	0.991 (0.457)	0.997 (0.446)	0.994 (0.336)	0.995 (0.486)	0.985 (0.375)
CC*	0.998 (0.792)	0.999 (0.785)	0.999 (0.709)	0.999 (0.809)	0.996 (0.738)
Reflections used in refinement	10271 (977)	12915 (1165)	9533 (929)	17030 (1685)	11115 (986)
Reflections used for R-free	1027 (97)	1293 (117)	955 (94)	1703 (169)	1111 (100)
R-work	0.2119 (0.3659)	0.1914 (0.2809)	0.1870 (0.3067)	0.1686(0.3028)	0.1758 (0.3019)
R-free	0.2468 (0.4084)	0.2366 (0.3072)	0.2390 (0.3425)	0.2006 (0.3423)	0.2182(0.3776)
No. of non-hydrogen atoms	2231	2336	2322	2450	2411
macromolecules	2197	2260	2252	2387	2373
ligands	28	59	59	8	8
solvent	6	17	11	55	30
Protein residues	272	280	280	305	305
RMS(bonds)	0.004	0.007	0.004	0.004	0.004
RMS(angles)	0.91	1.16	0.96	1.03	0.99
Ramachandran favored (%)	97.01	96.04	96.40	97.67	96.67
Ramachandran allowed (%)	2.99	3.60	3.24	2.33	2.67
Ramachandran outliers (%)	0.00	0.36	0.36	0.00	0.67
Rotamer outliers (%)	1.63	3.17	3.57	2.68	3.45
Clashscore	1.81	1.96	5.90	1.26	0.64
Average B-factor	89.01	70.17	67.02	56.68	54.75
macromolecules	89.15	70.48	67.24	56.71	54.82

ligands	80.62	59.40	60.38	103.82	79.82
solvent	78.31	65.59	58.47	48.56	42.54
Coordinate error (Å)	0.464	0.297	0.385	0.276	0.311
No. of TLS groups	3	2	4	6	6

Statistics for the highest-resolution shell are shown in parentheses.

Table S5 Released HP structures in the Protein Data Bank by January 2026 identified via the structure title search items “pressure”, “MPa” or “bar”. PDB entries are sorted by their deposition date starting with the most recent.

PDB ID	Deposition Date	Pressure [MPa]	Collection Temp.[K]	Exp. Method	Structure Title
9PL1	2025-07-15	280	298	NMR	Structure of metastable folding intermediate of V17A/V26A ubiquitin derived from pressure-jump NMR data
9C8O	2024-06-12	150	293	X-ray	High-resolution structure of cytochrome c peroxidase from yeast at ambient temperature and 1.5 kbar
9C8P	2024-06-12	300	293	X-ray	High-resolution structure of cytochrome c peroxidase from yeast at ambient temperature and 3.0 kbar
8Y23	2024-01-25	?	100	X-ray	X-ray crystal structure of ALiS4-Streptavidine complex without cryo-protectant using a high-pressure cryocooling method
8Y24	2024-01-25	?	100	X-ray	X-ray crystal structure of ALiS4-Streptavidine complex with 10% glycerol using a high-pressure cryocooling method
8Y25	2024-01-25	?	100	X-ray	X-ray crystal structure of ALiS4-Streptavidine complex with 20% glycerol using a high-pressure cryocooling method
8Y26	2024-01-25	?	100	X-ray	X-ray crystal structure of ALiS5-Streptavidine complex without cryo-protectant using a high-pressure cryocooling method
8Y27	2024-01-25	?	100	X-ray	X-ray crystal structure of ALiS5-Streptavidine complex with 10% glycerol using a high-pressure cryocooling method
8Y28	2024-01-25	?	100	X-ray	X-ray crystal structure of ALiS5-Streptavidine complex with 20% glycerol using a high-pressure cryocooling method
8Y29	2024-01-25	?	100	X-ray	X-ray crystal structure of ALiS2-Streptavidine complex without cryo-protectant using a high-pressure cryocooling method

8Y2A	2024-01-25	?	100	X-ray	X-ray crystal structure of ALiS2-Streptavidine complex with 10% glycerol using a high-pressure cryocooling method
8Y2B	2024-01-25	?	100	X-ray	X-ray crystal structure of ALiS2-Streptavidine complex with 20% glycerol using a high-pressure cryocooling method
8OSM	2023-04-19	200	293	X-ray	GTPASE HRAS IN COMPLEX WITH ZN-CYCLEN AT 200 MPA PRESSURE
8OSO	2023-04-19	500	293	X-ray	GTPase HRAS in complex with Zn-cyclen under 500 MPa pressure
8SLU	2023-04-24	205	100	X-ray	Crystal structure of human STEP (PTPN5) at cryogenic temperature (100K) and high pressure (205 MPa)
8CPC	2023-03-02	210	103	ED	3D electron diffraction structure of Hen Egg-White Lysozyme from nano-crystals obtained by high pressure freezing and cryo-sectioning
8BRZ	2022-11-24	52	295	X-ray	Room-temperature structure of *Pedobacter heparinus* *N*-acetylglucosamine 2- epimerase at 52 MPa helium gas pressure in a sapphire capillary
8BS0	2022-11-24	80	295	X-ray	Room-temperature structure of *Pedobacter heparinus* *N*-acetylglucosamine 2-epimerase at 80 MPa helium gas pressure in a sapphire capillary
8BS2	2022-11-24	104	295	X-ray	Room-temperature structure of SARS-CoV-2 Main protease at 104 MPa helium gas pressure in a sapphire capillary
8F2G	2022-11-08	440	298	X-ray	Crystal structure of Hen Egg White Lysozyme at 0.44 GPa
7ZQL	2022-05-01	310	298	X-ray	d(CGCGTTAACGCG)2 B-DODECAMER AT 3100 BARS (310 MPa)
7ZQM	2022-05-01	300	298	X-ray	d(CGCGCG)2 Z-DNA AT 3000 BARS (300 MPa)
7ZQN	2022-05-01	540	298	X-ray	d(CGCGCG)2 Z-DNA AT 5400 BARS (540 MPa)
7ZQO	2022-05-01	715	298	X-ray	d(CGCGCG)2 Z-DNA AT 7150 BARS (715 MPa)
7Q76	2021-11-09	22	295	X-ray	Room temperature structure of RNase A at 22 MPa helium gas pressure in a sapphire capillary

7Q77	2021-11-09	50	295	X-ray	Room temperature structure of RNase A at 50 MPa helium gas pressure in a sapphire capillary
7Q78	2021-11-09	72	295	X-ray	Room temperature structure of RNase A at 72 MPa helium gas pressure in a sapphire capillary
7Q79	2021-11-09	100	295	X-ray	Room temperature structure of RNase A at 100 MPa helium gas pressure in a sapphire capillary
7Q7A	2021-11-09	120	295	X-ray	Room temperature structure of RNase A at 120 MPa helium gas pressure in a sapphire capillary
7Q7D	2021-11-09	111	295	X-ray	Room temperature structure of the human Serine/Threonine Kinase 17B (STK17B/DRAK2) in complex with ATP/ADP at 111 MPa helium gas pressure in a sapphire capillary
7Q7G	2021-11-09	30	295	X-ray	Room temperature structure of the *Rhodobacter Sphaeroides* Photosynthetic Reaction Center F(M197)H mutant at 30 MPa helium gas pressure in a sapphire capillary
7Q7H	2021-11-09	51	295	X-ray	Room temperature structure of the *Rhodobacter Sphaeroides* Photosynthetic Reaction Center F(M197)H mutant at 51 MPa helium gas pressure in a sapphire capillary
7Q7J	2021-11-09	75	295	X-ray	Room temperature structure of the *Rhodobacter Sphaeroides* Photosynthetic Reaction Center F(M197)H mutant at 75 MPa helium gas pressure in a sapphire capillary
7Q7M	2021-11-09	100	295	X-ray	Room temperature structure of the *Rhodobacter Sphaeroides* Photosynthetic Reaction Center F(M197)H mutant at 100 MPa helium gas pressure in a sapphire capillary
7Q7N	2021-11-09	120	295	X-ray	Room temperature structure of the *Rhodobacter Sphaeroides* Photosynthetic Reaction Center F(M197)H mutant at 120 MPa helium gas pressure in a sapphire capillary
7Q09	2021-10-14	150	100	X-ray	URATE OXIDASE AZA-XANTHINE COMPLEX UNDER 1500 BAR OF ARGON
7PWN	2021-10-07	100	100	X-ray	URATE OXYDASE AZA-XANTHINE COMPLEX AT 1000 BARS (100 MPa) OF ARGON

7PUF	2021-09-29	60	100	X-ray	urate oxidase azaxanthine complex under 600 bar (60 MPa) of argon
7P0C	2021-06-29	210	293	X-ray	URATE OXIDASE WITH 8-AZAXANTHINE UNDER 210 MPA PRESSURE
7P0D	2021-06-29	310	293	X-ray	URATE OXIDASE WITH 8-AZAXANTHINE UNDER 310 MPA PRESSURE
7OGA	2021-05-06	200	293	X-ray	GTPase HRAS under 200 MPa pressure
7OGB	2021-05-06	500	293	X-ray	GTPase HRAS under 500 MPa pressure
7OGC	2021-05-06	650	293	X-ray	GTPase HRAS under 650 MPa pressure
7OGE	2021-05-06	200	293	X-ray	GTPase HRAS mutant D33K under 200 MPa pressure
7OGF	2021-05-06	900	293	X-ray	GTPase HRAS mutant D33K under 900 MPa pressure
6ZMC	2020-07-02	200	100	X-ray	Structure of the tRNA-Monooxygenase enzyme MiaE frozen under 2000 bar using the high pressure freezing method
6TEF	2019-11-11	0.5	100	X-ray	Crystal structure of monooxygenase RutA complexed with dioxygen under 0.5 MPa / 5 bars of oxygen pressure.
6TEG	2019-11-11	1.5	100	X-ray	Crystal structure of monooxygenase RutA complexed with uracil and dioxygen under 1.5 MPa / 15 bars of oxygen pressure.
6SP8	2019-08-31	15	100	X-ray	Structure of hyperstable haloalkane dehalogenase variant DhaA115 prepared by the 'soak-and-freeze' method under 150 bar of krypton pressure
6SGG	2019-08-04	1.5	100	X-ray	Crystal structure of monooxygenase RutA complexed with dioxygen under 1.5 MPa / 15 bars of oxygen pressure.
6RGM	2019-04-17	13	100	X-ray	urate oxidase under 130 bar of krypton
6QQG	2019-02-18	60	273	X-ray	High pressure structure of bovine insulin (60 MPa)
6QRK	2019-02-19	200	293	X-ray	High pressure structure of bovine insulin (200 MPa)
6QRH	2019-02-19	100	273	X-ray	High pressure structure of bovine insulin (100 MPa)
6IC1	2018-12-01	0.9	100	X-ray	urate oxidase under 90 bar of krypton
6I9Z	2018-11-26	6.5	291	X-ray	urate oxidase under 65 bar of argon
6I9X	2018-11-26	3.5	291	X-ray	urate oxidase under 35 bar of argon

6IA1	2018-11-26	12	100	X-ray	urate oxidase under 120 bar of argon
6IA3	2018-11-26	22	100	X-ray	urate oxidase under 220 bar (22 MPa) of argon
6IA9	2018-11-26	220	100	X-ray	urate oxidase under 2000 bar (220 MPa) of argon
6GDD	2018-04-23	120	298	X-ray	DIHYDROOROTASE FROM AQUIFEX AEOLICUS UNDER 1200 BAR OF HYDROSTATIC PRESSURE
6GDF	2018-04-23	60	298	X-ray	DIHYDROOROTASE FROM AQUIFEX AEOLICUS UNDER 600 BAR OF HYDROSTATIC PRESSURE
6FY9	2018-03-11	1.5	100	X-ray	The crystal structure of EncM complex with xenon under 15 bars Xe pressure
6FYC	2018-03-11	1.5	100	X-ray	The crystal structure of EncM L144M mutant complex with dioxygen under 15 bars O2 pressure
6FP3	2018-02-09	0.5	100	X-ray	The crystal structure of EncM complexed with dioxygen under 5 bar of oxygen pressure.
6FOQ	2018-02-08	1.5	100	X-ray	The crystal structure of EncM complexed with dioxygen under 15 bar of oxygen pressure.
6FOW	2018-02-08	1	100	X-ray	The crystal structure of EncM complexed with dioxygen under 10 bar of oxygen pressure.
5Z6J	2018-01-23	220	297	X-ray	High-pressure Crystal Structure Analysis of M20 loop closed DHFR at 220 MPa
5Z6K	2018-01-23	400	297	X-ray	High-pressure Crystal Structure Analysis of M20 loop closed DHFR at 400 MPa
5Z6L	2018-01-23	650	297	X-ray	High-pressure Crystal Structure Analysis of M20 loop closed DHFR at 650 MPa
5Z6M	2018-01-23	800	297	X-ray	High-pressure Crystal Structure Analysis of M20 loop closed DHFR at 800 MPa
6EYE	2017-11-12	15	100	X-ray	Crystal structure of murine neuroglobin under 150 bar krypton
6B7V	2017-10-05	120	100	X-ray	Structure of hen egg-white lysozyme pre-treated with high-pressure homogenization at 120 MPa
6B7W	2017-10-05	600	100	X-ray	Structure of hen egg-white lysozyme pre-treated with high pressure (600 MPa) under isobaric condition
6AYK	2017-09-08	1.2	100	X-ray	Crystal structure of TEM1 beta-lactamase mutant I263A in the presence of 1.2 MPa xenon

5O6N	2017-06-07	220	100	X-ray	High pressure flash cooled concanavalin A
5O6Q	2017-06-07	220	100	X-ray	High pressure flash cooled hen egg white lysozyme
5O27	2017-05-19	3	293	X-ray	Crystal structure of murine neuroglobin mutant V140W under 30 bar xenon pressure
5O17	2017-05-18	10	293	X-ray	Crystal structure of murine neuroglobin under 100 bar krypton
5O1K	2017-05-18	2	293	X-ray	Crystal structure of murine neuroglobin mutant V140W under 20 bar xenon pressure
5NW6	2017-05-05	5	293	X-ray	Crystal structure of murine neuroglobin under 50 bar krypton pressure
5NVI	2017-05-04	5	293	X-ray	Crystal structure of murine neuroglobin under 50 bar argon pressure
5MJC	2016-11-30	5	100	X-ray	metNeuroglobin under oxygen at 50 bar
5MJD	2016-11-30	8	100	X-ray	metNgb under oxygen at 80 bar
5T8B	2016-09-07	350	100	X-ray	Crystal structure of lpg1496 under 350 MPa
5T8C	2016-09-07	300	100	X-ray	Crystal structure of lpg1496 under 300 MPa
5T8L	2016-09-07	350	100	X-ray	Crystal structure of Snf7 under 350 MPa
5T8N	2016-09-07	200	100	X-ray	Crystal structure of Snf7 under 200 MPa
5LKF	2016-07-22	550	298	X-ray	Bovine beta-lactoglobulin complex with myristic acid at high pressure (0.55 GPa)
5IO7	2016-03-08	430	298	X-ray	Bovine beta-lactoglobulin complex with dodecane at high pressure (0.43 GPa)
5KPU	2016-07-05	1.2	100	X-ray	Crystal structure of TEM1 beta-lactamase mutant I263L in the presence of 1.2 MPa xenon
5I63	2016-02-15	1.2	100	X-ray	Crystal structure of TEM1 beta-lactamase mutant I263N in the presence of 1.2 MPa xenon
5HW1	2016-01-28	1.2	100	X-ray	Crystal structure of TEM1 beta-lactamase in the presence of 1.2 MPa xenon
5FSS	2016-01-07	4	100	X-ray	Structure of thermolysin prepared by the 'soak-and-freeze' method under 40 bar of krypton pressure
5FST	2016-01-07	10	100	X-ray	Structure of lysozyme prepared by the 'soak-and-freeze' method under 100 bar of krypton pressure

5FSJ	2016-01-06	4.5	100	X-ray	Structure of thermolysin prepared by the 'soak-and-freeze' method under 45 bar of oxygen pressure
5FSP	2016-01-06	10	100	X-ray	Structure of thermolysin prepared by the 'soak-and-freeze' method under 100 bar of krypton pressure
5FRC	2015-12-16	4.2	100	X-ray	Structure of urate oxidase prepared by the 'soak-and-freeze' method under 42 bar of oxygen pressure
5F2A	2015-12-01	310	293	X-ray	Crystal structure of murine neuroglobin mutant F106W at 310 MPa pressure
5EOH	2015-11-10	270	293	X-ray	Crystal structure of murine neuroglobin at 270 MPa pressure
5EQM	2015-11-13	310	293	X-ray	Crystal structure of murine neuroglobin at 310 MPa pressure
5EV5	2015-11-19	150	293	X-ray	Crystal structure of murine neuroglobin mutant V101F at 150 MPa pressure
5EYJ	2015-11-25	240	293	X-ray	Crystal structure of murine neuroglobin mutant V101F at 240 MPa pressure
5F0B	2015-11-27	280	293	X-ray	Crystal structure of murine neuroglobin mutant F106W at 280 MPa pressure
5C2W	2015-06-16	2	100	X-ray	Kuenenia stuttgartiensis Hydrazine Synthase Pressurized with 20 bar Xenon
4XEN	2014-12-24	920	298	X-ray	High pressure protein crystallography of hen egg white lysozyme in complex with Tetra-*N*-acetylchitotetraose at 920 MPa
4X5G	2014-12-05	270	298	X-ray	ecDHFR complexed with folate and NADP+ at 270 MPa
4X5H	2014-12-05	500	298	X-ray	ecDHFR complexed with folate and NADP+ at 500 MPa
4X5I	2014-12-05	660	298	X-ray	ecDHFR complexed with folate and NADP+ at 660 MPa
4X5J	2014-12-05	750	298	X-ray	ecDHFR complexed with folate and NADP+ at 750 MPa
4WLT	2014-10-08	190	298	X-ray	High pressure protein crystallography of hen egg white lysozyme at 190 MPa

4WLX	2014-10-08	280	298	X-ray	High pressure protein crystallography of hen egg white lysozyme at 280 MPa
4WLY	2014-10-08	380	298	X-ray	High pressure protein crystallography of hen egg white lysozyme at 380 MPa
4WM1	2014-10-08	500	298	X-ray	High pressure protein crystallography of hen egg white lysozyme at 500 MPa
4WM2	2014-10-08	600	298	X-ray	High pressure protein crystallography of hen egg white lysozyme at 600 MPa
4WM3	2014-10-08	710	298	X-ray	High pressure protein crystallography of hen egg white lysozyme at 710 MPa
4WM4	2014-10-08	800	298	X-ray	High pressure protein crystallography of hen egg white lysozyme at 800 MPa
4WM5	2014-10-08	890	298	X-ray	High pressure protein crystallography of hen egg white lysozyme at 890 MPa
4WM6	2014-10-08	950	298	X-ray	High pressure protein crystallography of hen egg white lysozyme at 950 MPa
4NWE	2013-12-06	3	298	X-ray	Lysozyme UNDER 30 BAR PRESSURE OF NITROUS OXIDE
4NWH	2013-12-06	3	298	X-ray	Lysozyme UNDER 30 BAR PRESSURE OF XENON
4NXC	2013-12-09	3	298	X-ray	SPERM WHALE MYOGLOBIN UNDER 30 BAR NITROUS Oxide
4NXA	2013-12-09	3	298	X-ray	SPERM WHALE MYOGLOBIN UNDER XENON PRESSURE 30 Bar
4O4T	2013-12-19	3	291	X-ray	MURINE NEUROGLOBIN UNDER XENON PRESSURE 30 bar
4O4Z	2013-12-19	3	291	X-ray	MURINE NEUROGLOBIN UNDER 30 BAR PRESSURE NITROUS Oxide
3WDN	2013-06-19	170	90	X-ray	High-resolution X-ray crystal structure of bovine H-protein using a high-pressure cryocooling method
3VL2	2011-11-29	160	293	X-ray	3-isopropylmalate dehydrogenase from *Shewanella oneidensis* MR-1 at 160 MPa
3VL3	2011-11-29	340	293	X-ray	3-isopropylmalate dehydrogenase from *Shewanella oneidensis* MR-1 at 340 MPa

3VL4	2011-11-29	410	293	X-ray	3-isopropylmalate dehydrogenase from *Shewanella oneidensis* MR-1 at 410 MPa
3VL6	2011-11-29	580	293	X-ray	3-isopropylmalate dehydrogenase from *Shewanella oneidensis* MR-1 at 580 MPa
3VL7	2011-11-29	650	293	X-ray	3-isopropylmalate dehydrogenase from *Shewanella oneidensis* MR-1 at 650 MPa
4A7D	2011-11-14	210	100	X-ray	X-ray crystal structure of HEWL flash-cooled at high pressure
4A7E	2011-11-14	210	100	X-ray	X-ray crystal structure of porcine insulin flash-cooled at high pressure
3PLE	2010-11-15	0.5	298	X-ray	urate oxidase under 0.5 MPa / 5 bars pressure of equimolar mixture xenon: nitrous oxide
3PLG	2010-11-15	1	298	X-ray	urate oxidase under 1.0 MPa / 10 bars pressure of equimolar mixture xenon: nitrous oxide
3PLH	2010-11-15	1.5	298	X-ray	urate oxidase under 1.5 MPa / 15 bars pressure of equimolar mixture xenon: nitrous oxide
3PLI	2010-11-15	1.8	298	X-ray	Urate oxidase under 1.8 MPa / 18 bars pressure of equimolar mixture xenon: nitrous oxide
3PLJ	2010-11-15	3	298	X-ray	Urate oxidase under 3.0 MPa / 30 bars pressure of equimolar mixture xenon: nitrous oxide
3PLM	2010-11-15	2	298	X-ray	Urate oxidase under 2.0 MPa / 20 bars pressure of equimolar mixture xenon: nitrous oxide
3PKS	2010-11-12	1.5	298	X-ray	Urate oxidase under 1.5 MPa / 15 bars pressure of nitrous oxide
3PKT	2010-11-12	2	298	X-ray	Urate oxidase under 2 MPa / 20 bars pressure of nitrous oxide
3PKU	2010-11-12	1	298	X-ray	Urate oxidase under 1 MPa / 10 bars pressure of nitrous oxide
3PK3	2010-11-11	3	298	X-ray	urate oxidase under 3.0 MPa / 30 bars pressure of nitrous oxide
3PK4	2010-11-11	3.2	298	X-ray	Urate oxidase under 3.2 MPa / 32 bars pressure of xenon
3PK6	2010-11-11	0.2	298	X-ray	Urate oxidase under 0.2 MPa / 2 bars pressure of xenon

3PK8	2010-11-11	0.5	298	X-ray	Urate oxidase under 0.5 MPa / 5 bars pressure of nitrous oxide
3PKF	2010-11-11	0.2	298	X-ray	Urate oxidase under 0.2 MPa / 2 bars pressure of equimolar mixture of xenon and nitrous oxide
3PKG	2010-11-11	2	298	X-ray	Urate oxidase under 2 MPa / 20 bars pressure of xenon
3PKH	2010-11-11	1.5	298	X-ray	Urate oxidase under 1.5 MPa / 15 bars pressure of xenon
3PKK	2010-11-11	0.5	298	X-ray	Urate oxidase under 0.5 MPa / 5 bars pressure of xenon
3PKL	2010-11-11	0.8	298	X-ray	Urate oxidase under 0.8 MPa / 8 bars pressure of xenon
3PJK	2010-11-10	1	298	X-ray	Urate oxidase under 1.0 MPa / 10 bars pressure of xenon
3MOU	2010-04-23	?	100	X-ray	Characterization of the Inhibitor Binding Site of the Dehaloperoxidase-Hemoglobin from *Amphitrite ornata* using High-Pressure Xenon Derivatization
3I7W	2009-07-09	670	298	X-ray	High pressure structure of wild-type RNase A (0.67 GPa)
3I7X	2009-07-09	350	293	X-ray	High pressure structure of I106A RNase A variant (0.35 GPa)
3I7Y	2009-07-09	480	293	X-ray	High pressure structure of I106A variant of RNase A (0.48 GPa)
3HW7	2009-06-17	570	298	X-ray	High pressure (0.57 GPa) crystal structure of bovine copper, zinc superoxide dismutase at 2.0 angstroms
3GLN	2009-03-12	2	100	X-ray	Carbonmonoxy Ngb under Xenon pressure
3GKT	2009-03-11	4	100	X-ray	Crystal structure of murine neuroglobin under Kr pressure
3GK9	2009-03-10	2	100	X-ray	Crystal structure of murine Ngb under Xe pressure
2KF3	2009-02-11	3	298	NMR	Barnase, low pressure reference NMR structure
2KF4	2009-02-11	200	298	NMR	Barnase high pressure structure
2KF5	2009-02-11	3	298	NMR	Barnase bound to d(CGAC), low pressure
2KF6	2009-02-11	200	298	NMR	Barnase bound to d(CGAC) high pressure
3F2M	2008-10-30	150	298	X-ray	Urate oxidase complexed with 8-azaxanthine at 150 MPa

3DPX	2008-07-09	500	100	X-ray	Structure of the Yellow Fluorescent Protein Citrine Frozen at 5000 Atmospheres: Structure 26 in a Series of 26 High Pressure Structures
3DPZ	2008-07-09	400	100	X-ray	Structure of the Yellow Fluorescent Protein Citrine Frozen at 4000 Atmospheres Number 3: Structure 25 in a Series of 26 High Pressure Structures
3DQ1	2008-07-09	400	100	X-ray	Structure of the Yellow Fluorescent Protein Citrine Frozen at 4000 Atmospheres Number 2: Structure 24 in a Series of 26 High Pressure Structures
3DQ2	2008-07-09	400	100	X-ray	Structure of the Yellow Fluorescent Protein Citrine Frozen at 4000 Atmospheres Number 1: Structure 23 in a Series of 26 High Pressure Structures
3DQ3	2008-07-09	250	100	X-ray	Structure of the Yellow Fluorescent Protein Citrine Frozen at 2500 Atmospheres: Structure 22 in a Series of 26 High Pressure Structures
3DQ4	2008-07-09	200	100	X-ray	Structure of the Yellow Fluorescent Protein Citrine Frozen at 2000 Atmospheres Number 2: Structure 20 in a Series of 26 High Pressure Structures
3DQ5	2008-07-09	196	100	X-ray	Structure of the Yellow Fluorescent Protein Citrine Frozen at 1960 Atmospheres: Structure 19 in a Series of 26 High Pressure Structures
3DQ6	2008-07-09	192	100	X-ray	Structure of the Yellow Fluorescent Protein Citrine Frozen at 1920 Atmospheres Number 2: Structure 18 in a Series of 26 High Pressure Structures
3DQ7	2008-07-09	192	100	X-ray	Structure of the Yellow Fluorescent Protein Citrine Frozen at 1920 Atmospheres Number 1: Structure 17 in a Series of 26 High Pressure Structures
3DQ8	2008-07-09	150	100	X-ray	Structure of the Yellow Fluorescent Protein Citrine Frozen at 1500 Atmospheres Number 2: Structure 16 in a Series of 26 High Pressure Structures
3DQ9	2008-07-09	150	100	X-ray	Structure of the Yellow Fluorescent Protein Citrine Frozen at 1500 Atmospheres Number 1: Structure 15 in a Series of 26 High Pressure Structures
3DQA	2008-07-09	125	100	X-ray	Structure of the Yellow Fluorescent Protein Citrine Frozen at 1250 Atmospheres Number 4: Structure 14 in a Series of 26 High Pressure Structures

3DQC	2008-07-09	125	100	X-ray	Structure of the Yellow Fluorescent Protein Citrine Frozen at 1250 Atmospheres Number 3: Structure 13 in a Series of 26 High Pressure Structures
3DQD	2008-07-09	125	100	X-ray	Structure of the Yellow Fluorescent Protein Citrine Frozen at 1250 Atmospheres Number 2: Structure 12 in a Series of 26 High Pressure Structures
3DQE	2008-07-09	125	100	X-ray	Structure of the Yellow Fluorescent Protein Citrine Frozen at 1250 Atmospheres Number 1: Structure 11 in a Series of 26 High Pressure Structures
3DQF	2008-07-09	100	100	X-ray	Structure of the Yellow Fluorescent Protein Citrine Frozen at 1000 Atmospheres Number 6: Structure 10 in a Series of 26 High Pressure Structures
3DQH	2008-07-09	100	100	X-ray	Structure of the Yellow Fluorescent Protein Citrine Frozen at 1000 Atmospheres Number 5: Structure 9 in a Series of 26 High Pressure Structures
3DQI	2008-07-09	100	100	X-ray	Structure of the Yellow Fluorescent Protein Citrine Frozen at 1000 Atmospheres Number 4: Structure 8 in a Series of 26 High Pressure Structures
3DQJ	2008-07-09	100	100	X-ray	Structure of the Yellow Fluorescent Protein Citrine Frozen at 1000 Atmospheres Number 3: Structure 7 in a Series of 26 High Pressure Structures
3DQK	2008-07-09	100	100	X-ray	Structure of the Yellow Fluorescent Protein Citrine Frozen at 1000 Atmospheres Number 2: Structure 6 in a Series of 26 High Pressure Structures
3DQL	2008-07-09	100	100	X-ray	Structure of the Yellow Fluorescent Protein Citrine Frozen at 1000 Atmospheres Number 1: Structure 5 in a Series of 26 High Pressure Structures
3DQM	2008-07-09	75	100	X-ray	Structure of the Yellow Fluorescent Protein Citrine Frozen at 750 Atmospheres: Structure 4 in a Series of 26 High Pressure Structures
3DQN	2008-07-09	50	100	X-ray	Structure of the Yellow Fluorescent Protein Citrine Frozen at 500 Atmospheres: Structure 3 in a Series of 26 High Pressure Structures
3DQU	2008-07-09	200	100	X-ray	Structure of the Yellow Fluorescent Protein Citrine Frozen at 2000 Atmospheres Number 1: Structure 20 in a Series of 26 High Pressure Structures

2ZKA	2008-03-13	1	277	X-ray	Urate oxidase complexed with 8-azaxanthine under 1.0 MPa oxygen pressure
2ZKB	2008-03-14	2.5	277	X-ray	Urate oxidase complexed with 8-azaxanthine under 2.5 MPa oxygen pressure
3CKS	2008-03-17	4	277	X-ray	Urate oxidase complexed with 8-azaxanthine under 4.0 MPa oxygen pressure
3BVD	2008-01-07	0.7	100	X-ray	Structure of Surface-engineered Cytochrome ba3 Oxidase from Thermus thermophilus under Xenon Pressure, 100psi 5min
2ZCC	2007-11-08	50	100	X-ray	Ubiquitin crystallized under high pressure
2VAH	2007-08-31	3	305	NMR	Solution structure of a B-DNA hairpin at low pressure.
2VAI	2007-08-31	200	305	NMR	Solution structure of a B-DNA hairpin at high pressure
2PL4	2007-04-19	550	295	X-ray	D-(GGTATACC) under 0.55 GPa hydrostatic pressure
2PL8	2007-04-19	1040	295	X-ray	D(GTATACC) under hydrostatic pressure of 1.04 GPa
2PLB	2007-04-19	1390	295	X-ray	D(GTATACC) under hydrostatic pressure of 1.39 GPa
2OQU	2007-02-01	145	110	X-ray	High Pressure Cryocooling of Capillary Sample Cryoprotection and Diffraction Phasing at Long Wavelengths (Elastase)
2OQN	2007-01-31	170	110	X-ray	High Pressure Cryocooling of Capillary Sample Cryoprotection and Diffraction Phasing at Long Wavelengths (Thaumatin)
2IE6	2006-09-18	2	277	X-ray	Annexin V under 2.0 MPa pressure of xenon
2IE7	2006-09-18	2	277	X-ray	Annexin V under 2.0 MPa pressure of nitrous oxide
2ICQ	2006-09-13	2	277	X-ray	urate oxidase under 2.0 MPa pressure of nitrous oxide
2IC0	2006-09-12	2	277	X-ray	Urate oxidase under 2.0 MPa pressure of xenon
2J52	2006-09-11	3	287	NMR	Solution Structure of GB1 domain Protein G and low and high pressure.
2J53	2006-09-11	200	287	NMR	Solution Structure of GB1 domain Protein G and low and high pressure.
2DKI	2006-04-11	1.2	100	X-ray	Crystal structure of 3-hydroxybenzoate hydroxylase from Comamonas testosteroni, under pressure of xenon gas (12 atm)
2FUB	2006-01-26	140	293	X-ray	Crystal structure of urate oxidase at 140 MPa

2B6T	2005-10-03	200	293	X-ray	T4 Lysozyme mutant L99A at 200 MPa
2B6W	2005-10-03	200	293	X-ray	T4 Lysozyme mutant L99A at 200 MPa
2B6X	2005-10-03	200	293	X-ray	T4 Lysozyme mutant L99A at 200 MPa
2B72	2005-10-03	100	293	X-ray	T4 Lysozyme mutant L99A at 100 MPa
2B73	2005-10-03	100	293	X-ray	T4 Lysozyme mutant L99A at 100 MPa
2B74	2005-10-03	100	293	X-ray	T4 Lysozyme mutant L99A at 100 MPa
2B75	2005-10-03	150	293	X-ray	T4 Lysozyme mutant L99A at 150 MPa
1W3X	2004-07-20	2	100	X-ray	Isopenicillin N synthase d-(L-a-aminoadipoyl)-(3R)-methyl-L-cysteine D-a-hydroxyisovaleryl ester complex (Oxygen exposed 5 minutes 20 bar)
1U0X	2004-07-14	1.4	100	X-ray	Crystal structure of nitrophorin 4 under pressure of xenon (200 psi)
1V80	2003-12-27	3	293	NMR	Solution structures of ubiquitin at 30 bar and 3 kbar
1V81	2003-12-27	300	293	NMR	Solution structures of ubiquitin at 30 bar and 3 kbar
1OA6	2003-01-02	200	309	NMR	The solution structure of bovine pancreatic trypsin inhibitor at high pressure
1LR3	2002-05-14	150	100	X-ray	Crystal structure of thaumatin prepared at high hydrostatic pressure
1GXX	2002-04-15	200	298	NMR	Solution structure of lysozyme at low and high pressure
1JP8	2001-08-01	150	295	X-ray	Sperm Whale met-Myoglobin (room temperature; high pressure)
1JP9	2001-08-01	200	95	X-ray	Sperm Whale met-Myoglobin (low temperature; high pressure)
1JPB	2001-08-01	200	95	X-ray	Sperm Whale met-Myoglobin (low temperature; high pressure)
1C3L	1999-07-28	0.8	278	X-ray	SUBTILISIN-CARLSBERG COMPLEXED WITH XENON (8 BAR)
1C1M	1999-07-22	0.8	277	X-ray	PORCINE ELASTASE UNDER XENON PRESSURE (8 BAR)
1C10	1999-07-16	0.8	277	X-ray	CRYSTAL STRUCTURE OF HEW LYSOZYME UNDER PRESSURE OF XENON (8 BAR)
1QTK	1999-06-28	5.5	277	X-ray	CRYSTAL STRUCTURE OF HEW LYSOZYME UNDER PRESSURE OF KRYPTON (55 BAR)

2LYM	1987-06-08	100	?	X-ray	CRYSTAL STRUCTURE OF HEN EGG-WHITE LYSOZYME AT A HYDROSTATIC PRESSURE OF 1000 ATMOSPHERES
3LYM	1987-06-08	100	?	X-ray	CRYSTAL STRUCTURE OF HEN EGG-WHITE LYSOZYME AT A HYDROSTATIC PRESSURE OF 1000 ATMOSPHERES
